# Brain structural covariances in the ageing brain in the UK Biobank

**DOI:** 10.1007/s00429-024-02794-4

**Published:** 2024-04-16

**Authors:** Chao Dong, Anbupalam Thalamuthu, Jiyang Jiang, Karen A. Mather, Perminder S. Sachdev, Wei Wen

**Affiliations:** 1 Centre for Healthy Brain Ageing (CHeBA), Discipline of Psychiatry and Mental Health, School of Clinical Medicine, UNSW, Sydney, Australia; 2 Neuropsychiatric Institute (NPI), Prince of Wales Hospital, Randwick, NSW 2031 Australia

**Keywords:** Brain ageing, Structural MRI, Structural covariance, Cognition

## Abstract

The morphologic properties of brain regions co-vary or correlate with each other. Here we investigated the structural covariances of cortical thickness and subcortical volumes in the ageing brain, along with their associations with age and cognition, using cross-sectional data from the UK Biobank (*N* = 42,075, aged 45–83 years, 53% female). As the structural covariance should be estimated in a group of participants, all participants were divided into 84 non-overlapping, equal-sized age groups ranging from the youngest to the oldest. We examined 84 cortical thickness covariances and subcortical covariances. Our findings include: (1) there were significant differences in the variability of structural covariance in the ageing process, including an increased variance, and a decreased entropy. (2) significant enrichment in pairwise correlations between brain regions within the occipital lobe was observed in all age groups; (3) structural covariance in older age, especially after the age of around 64, was significantly different from that in the youngest group (median age 48 years); (4) sixty-two of the total 528 pairs of cortical thickness correlations and 10 of the total 21 pairs of subcortical volume correlations showed significant associations with age. These trends varied, with some correlations strengthening, some weakening, and some reversing in direction with advancing age. Additionally, as ageing was associated with cognitive decline, most of the correlations with cognition displayed an opposite trend compared to age associated patterns of correlations.

**Supplementary Information:**

The online version contains supplementary material available at 10.1007/s00429-024-02794-4.

## Introduction

Morphological properties of brain regions co-vary with each other, which can be modelled by brain structural covariance (Mechelli et al. 2005). The simplest way of defining structural covariance is to estimate the correlation between the morphological property of two brain regions at the group-level. The “structural covariance matrix” refers to the correlation matrix of every pair of brain regions under examination (Carmon et al. 2020). Cortical thickness and subcortical volumes are sensitive to certain types of changes in the brain, such as changes related to ageing, or disease. For example, cortical thickness tends to decrease with age (Fjell et al. 2009), and subcortical volumes can be affected by diseases such as Alzheimer’s (de Jong et al. 2008) and Parkinson’s (Tinaz et al. 2011). The examination of these structural covariances in the ageing brain will add to our understanding of brain structure and age-related diseases (Montembeault et al. 2016; Nestor et al. 2017).

It has been increasingly recognised that brain structural covariance reflects a synchronised maturational process during early childhood and adolescence, as well as coordinated atrophy and decline in ageing brains (Alexander-Bloch et al. 2013). To study age-related differences in structural covariance, researchers have employed a range of methodologies. For instance, a previous study explored age-related differences in grey matter density structural covariance in the different developmental stages of childhood and adolescence by dividing their participants into four equally sized age groups (Zielinski et al. 2010). Structural covariances were also compared among four middle-aged groups (Hafkemeijer et al. 2014) as well as six age groups across the life span (DuPre and Spreng 2017), showing significant age-related differences. In addition, some studies applied sliding-window age configurations, indicating a mixture of linear and non-linear alterations in cortical thickness covariance during maturation (Váša et al. 2018; Vijayakumar et al. 2021). However, much remains to be learned about the structural covariance in the ageing process, as the majority of studies have focused on brain development during childhood and adolescence (Sotiras et al. 2017) and have generally been limited by small sample sizes and low statistical power.

Structural covariance changes with age, while cognition usually declines during the ageing process (Bishop et al. 2010; Grady 2012). Compared with young adults, older adults showed reduced structural covariance in brain networks that sustain high-order cognitive function (Montembeault et al. 2012). Structural covariance was also correlated with general cognitive functions (Spreng and Gary 2013). However, the details of associations between structural covariance and cognition remains unclear.

In this study, we aimed to investigate the associations between brain structural covariances (cortical thickness and subcortical volumes), age, and cognition in a large population sample of middle-aged and older adults from the UK Biobank. As structural covariances need to be estimated at the group level, we divided our sample into age-specific groups. Within each age group, we then estimated the covariances in cortical thickness and in subcortical volume separately. Here, we hypothesised that structural covariance would show age-related properties and would be also associated with cognition.

## Methods

### Participants

All our data were drawn from the UK Biobank, which is a population-based study consisting of over 500,000 participants aged between 40 and 70 years at study entry (Sudlow et al. 2015). Written consent was acquired from all participants and ethics approval was provided by the National Health Service National Research Ethics Service (11/NW/0382). Imaging data and cognition data in the current study were acquired at the first imaging assessment visit (instance 2). Here we used the MRI data released in 2021 under the UK Biobank application number 37,103. In the current study, 42,075 participants were included in the final analysis. The age range of the participants was 45 to 83 years with the mean of 64.5 years.

### Neuroimaging measures

Brain anatomical measures of cortical thickness and subcortical volumes were derived from structural MRI scans (Siemens Skyra 3T with a standard Siemens 32-channel RF receive head coil). The sequence parameters of T1-weighted structural imaging were: spatial resolution 1 × 1 × 1 mm; field of view 208 × 256 × 256 matrix; TI (inversion time) 880 ms; TR (repetition time) 2000 ms. The full protocol is available at http://biobank.ctsu.ox.ac.uk/crystal/refer.cgi?id=2367. We used data that were preprocessed by UK Biobank. Brain cortical parcellation was based on Desikan-Killiany atlas (Desikan et al. 2006) and performed by FreeSurfer (Fischl 2012). We used cortical thickness measures of 33 cortical regions, seven subcortical volumetric measures, intracranial volume (ICV), and global mean cortical thickness for each hemisphere generated by FreeSurfer. The whole brain mean thickness was calculated as the average between the left and right mean thickness. The outliers were defined as the MRI measures which were greater than five standard deviations from the mean. Individuals with any measure as an outlier were then removed from the study.

### Cognition

Cognitive assessments were administered on a fully-automated touchscreen questionnaire (Sudlow et al. 2015). Seven tests from the UK Biobank battery of cognitive tests were selected for the current study to represent three cognitive domains: processing speed, executive function, and memory. The details of cognitive tests can be found in (Du et al. 2021). Specifically, “Reaction Time”, “Trail Making Test A”, and “Symbol Digit Substitution” formed the Processing Speed domain; “Numeric Memory” and “Pairs Matching” contributed to the Memory domain; and “Trail Making Test B” and “Fluid Intelligence” formed the Executive Function domain. Therefore, three cognitive domains (processing speed, memory, and executive function) were then used in our study.

All the raw test scores were first transformed to z-scores by using the mean and standard deviation of a healthy reference sample (Du et al. 2021). Global cognition scores were computed in a similar way by averaging these domain scores and then transforming to z-scores. There were 27,140 participants with both MRI scans and full cognition data.

### Statistical analyses

#### Construction of structural covariance in each age-specific group

Regional cortical thickness (*n* = 33) and subcortical volumes (*n* = 7) were defined as the average of the left and right hemispheres. Covariances are estimated by all pairwise correlations, so these two terms are used interchangeably in the current study. The followings were the analysis of covariance matrices across age groups:

1. All 42,075 participants were sorted according to age from the youngest to the oldest, and were then split into nonoverlapping, equal-sized 84 groups with approximately 500 participants in each group (the last age group included 575 participants).
2. We obtained residuals of regional cortical thickness estimates after regressing out sex, scanner, and global mean cortical thickness, and then calculated within each group pairwise Pearson correlations across all the 33 regions of interest (ROIs), generating a 33 × 33 correlation matrix map for each age group which contained (33 × 32)/2 = 528 pairwise correlations. Controlling for global mean cortical thickness helps to account for overall differences in cortical thickness between individuals, focusing specifically on the patterns of covariance that are independent of these global differences.
3. Similarly, residuals for the subcortical volume estimates were obtained after regressing out sex, scanner, and intracranial volume (ICV), and a 7 × 7 Pearson correlation matrix in each group [(7 × 6)/2 = 21 pairwise correlations] was then generated.
4. To investigate if the structural covariance was different by sex, we also estimated cortical thickness and subcortical volume covariances for the same 84 groups in males and females separately without regressing out sex when computing residuals.

All cortical and subcortical regions can be found in Table S1 and Table S2.

#### Whole brain variability properties of structural covariance across all age groups

To examine the age-related differences of the overall correlation matrices, we have computed three whole brain variability measures: (i) variance; (ii) von Neumann entropy; and (iii) proportion of pairs of correlations that differ significantly within the group. The sample variance of whole pairwise correlations captured the variability of the correlation coefficients. Entropy, as a measure of brain complexity, plays an important role in quantifying brain’s capacity for adaptation and characterising the brain functions. Functional MRI studies have shown that the entropy decreases during the ageing process (Cieri et al. 2021; Jia et al. 2017). We have applied von Neumann entropy measure to the structural brain covariances. We also have computed the proportion of significant pairwise differences within the correlation matrix of each of the age groups. This proportion captures differences in regional correlation coefficients, and it may be different across the age groups. These three variability measures for each of age group correlation matrices were examined against median age.

Let R denote the Pearson correlation matrix of the cortical thickness (33 × 33) or subcortical volumes (7 × 7). The three measures were calculated as follows:

##### Variance

the sample variance of the lower diagonal elements of the R matrix.

**Von Neumann entropy**

$$S\left(\rho \right)=-Trace\left(\rho \,\text{log}\left(\rho \right)\right)=-\sum{\lambda_{j}}\text{log}(\lambda_{j}),\,\rho=\frac{R}{N}$$

where N is the number of ROIs in the correlation matrix (*N* = 33 or 7) and ${\lambda }_{j}$ are the eigen values of the $\rho$ matrix (Felippe et al. 2021). The entropy takes minimum value when all the correlation coefficients are unity and reaches the maximum value (log N) if the all the correlations are zero.

##### Proportion

To examine the pair-wise correlation differences with each group, a test for equality of pairs of correlations was performed. The proportions of pairs that differ significantly out of the total number of comparisons (total number of tests = 528 × 527/2 = 139,128 for cortical regions and 21 × 20/2 = 210 for subcortical regions) were computed from the correlation matrix of each age group. Tests for equality of two dependent correlations measured on the same set of individuals were performed using the Steiger’s method (Steiger 1980) as implemented in the R package *cocor* (Diedenhofen and Musch 2015).

In order to investigate if there are sex differences in these 3 measures (variance measure, von Neumann entropy and the proportion of pairs that differ significantly), the differences between males and females on these 3 measures across 84 age groups were assessed using an independent samples t-test.

#### Enrichment of correlation coefficients between and within lobar regions

To test for the enrichment of correlation coefficients between and within lobar regions, Over-representation of correlation analysis (ORCA) was performed (Pomyen et al. 2015). ORCA is a method to test whether greater numbers of significant correlations exist between two brain regions than expected by chance. Here four lobes were included: frontal, temporal, parietal, and occipital (Table S1). Significant enrichment of correlation coefficients above a threshold than by chance between/within lobe regions was assessed using the hypergeometric probability distribution. The correlation threshold was determined using the maximum Shannon entropy measure (Shannon 1948). ORCA was performed within each lobe and between all possible pairs of lobes (4 × 3/2 = 6) for the correlation matrix of each age group. Bonferroni correction was used to adjust for multiple hypotheses testing (*n* = 10; 4 within lobe + 6 all possible pairs of lobes).

#### Test for equality of correlation coefficients between the youngest age group versus each of the other groups

Global tests for equality of elements between two independent correlation matrices [the youngest age group (group 1) and each of other groups (group 2-group 84)] were done using the “pattern hypothesis” approach (Steiger 1980) as implemented in the R application MML-WBCORR (Fouladi 2018). The test of equality of elements of correlations in the first age group was compared with corresponding elements of the correlation matrices of the other age groups. Similarly, the elements of correlation matrices obtained from using male and female samples separately at each age group was compared.

#### Associations between structural covariance and age

To test the associations between pairwise correlations and age, we estimated the associations between each element of the correlation matrix and age (median age in each group) across all 84 groups by using Pearson’s correlation. Specifically, we firstly applied Fisher’s r-to-z transformation to all elements of the correlation matrix and then tested the association between each Fisher’s z-transformed correlation and age across all 84 correlation matrices. Therefore, (33 × 32)/2 = 528 associations of cortical thickness correlations and (7 × 6)/2 = 21 associations of subcortical volume correlations were estimated. Similarly, we tested the associations between Fisher’s z-transformed correlation and median cognitive performance (processing speed, executive function, memory, and global cognition) across all 84 groups. As some participants did not have cognitive data, they were excluded from the analysis and the median cognition was estimated for each group using only the participants with available data. Sex and scanner covariates for this analysis were not used because the correlation matrices were generated after removing the effects of these covariates. For all analyses, a Bonferroni-corrected p-value < 0.05 (for example, p-value threshold for cortical thickness correlation is *p* < 0.05/528 = 9.47e-05; and for subcortical correlation: *p* < 0.05/21 = 0.0024) was considered statistically significant. Statistical analyses were performed using R version 4.1.0.

#### Additional analysis

In the current study, 33 cortical regions in each hemisphere are symmetric. Averaging brain ROIs from both hemispheres can simplify the analysis and interpretation. However, to provide comprehensive understanding of structural covariance, we also estimated structural covariances from left and right hemispheres separately. Furthermore, as global mean cortical thickness has major effects on cortical thickness covariance, we also conducted covariance analysis without removing global mean cortical thickness in Supplementary.

We further examined the associations between structural covariance and age by dividing all participants into 140 groups, with approximately 300 participants in each group, as well as 52 groups with approximately 800 participants in each group. This approach allowed for a robust validation of the grouping strategy.

## Results

### Sample characteristics and experimental design

**Fig. 1:**
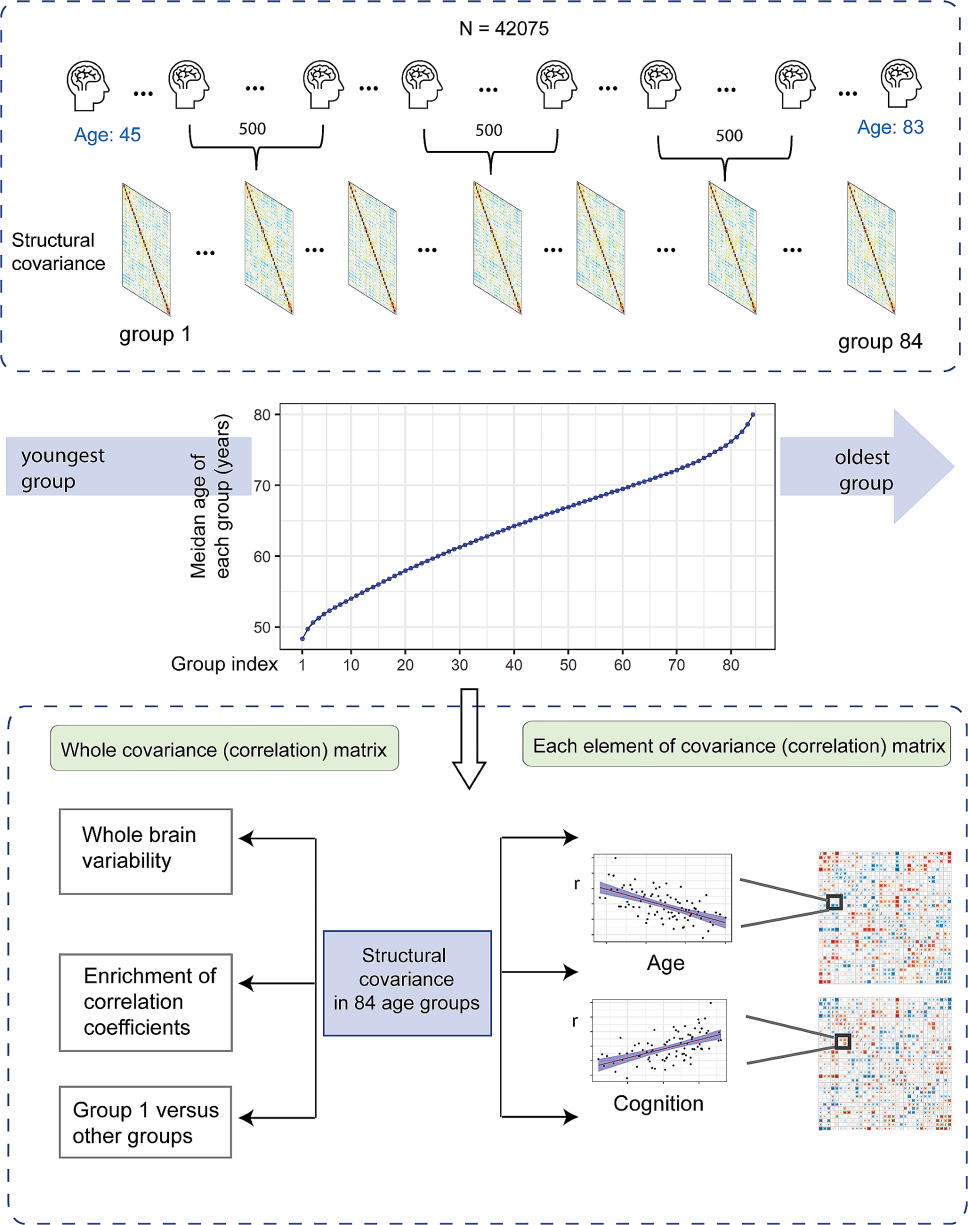
Conceptual overview of the study. Firstly, all 42,075 participants were ordered from the youngest to the oldest. They were then divided into equally sized 84 groups with 500 participants in each group. Structural covariance was estimated in each group. Secondly, we explored these structural covariances from the whole covariance (correlation) matrix level and each element of covariance (correlation) matrix level. For the whole matrix level, whole brain variability properties were estimated across all age groups. We also performed over-representation of correlation analysis (ORCA) to test the enrichment of correlation coefficients between and within brain lobar regions. Additionally, we compared the covariance of the first group with those in other groups. For the matrix element level, we calculated the associations between structural covariance, age, and cognition, obtaining two association matrices

Sample characteristics are shown in Table 1, and the conceptual overview of the study is depicted in (Fig. 1). Firstly, the structural covariance (correlation) matrices were established in each group. The cortical structural covariance of the first two youngest groups (group 1, 2) and last two oldest groups (group 83, 84) can be found in (Fig. S1). Then the analyses of whole covariance (correlation) matrix and each element of covariance (correlation) matrix were performed. Details can be found in Fig. 1.

**Table 1:** Sample characteristics in UK Biobank (instance 2)

*Participants with MRI data*		
	Size	Mean age ± SD (age range)
All participants	*N* = 42,075	64.5 ± 7.7 (44.6–82.8)
Male	*N* = 19,752 (46.9%)	65.2 ± 7.8 (44.6–82.5)
Female	*N* = 22,323 (53.1%)	63.8 ± 7.5 (45.2–82.8)
*Participants with both MRI and cognition data*		
All participants	*N* = 27,140	64.7 ± 7.6 (47.0-82.8)

Instance 2 means the first imaging assessment. SD: standard deviation

### Whole brain variability properties of structural covariance across age groups

**Fig. 2:**
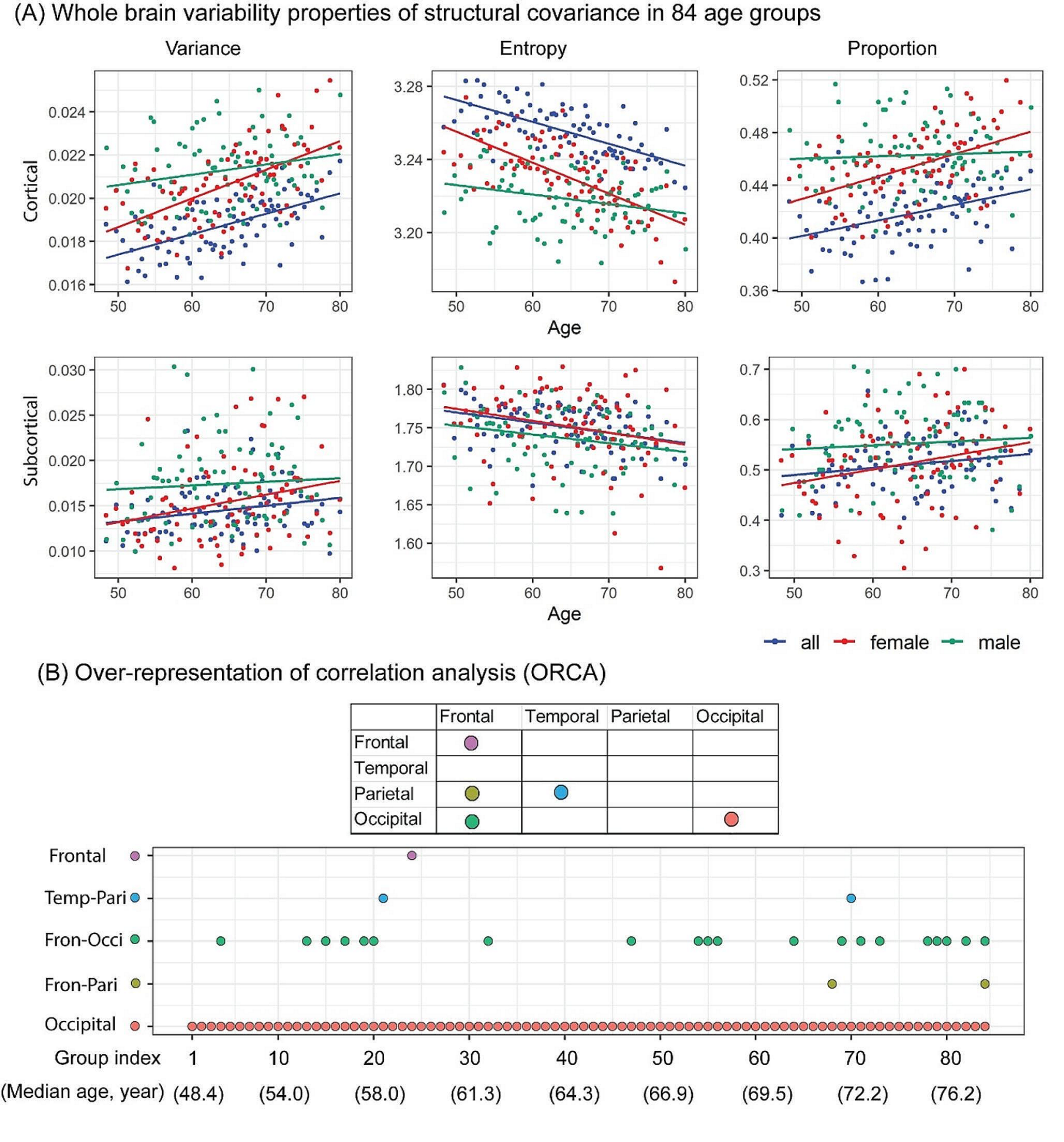
(**A**) Whole brain variability properties of structural covariance in 84 age groups. Three global measures: variance, entropy, and proportions of pairs that differ significantly were estimated both in cortical thickness covariance and subcortical volume covariance. The points represent the corresponding measure in each age-group. Here, male, female, and the combined participants were investigated separately. (**B**) Over-representation of correlation analysis (ORCA). The x-axis represents 84 age groups, and the dots located in different positions indicate that significant enrichment of correlation coefficients between or within lobes were found in the specific age groups (Bonferroni correction). Note that more correlations existed within occipital lobe than expected by chance, and this phenomenon was stable across all age groups. Significant enrichment of pairwise correlations was also observed between brain regions in frontal and occipital lobe

For the cortical thickness covariance, the variance of whole brain pair-wise correlations increased significantly with age (*r* = 0.56, *p* = 2.17e-10), and the proportion of pairs that differed significantly also increased with age (*r* = 0.40, *p* = 1.65e-04). Entropy decreased significantly with age (*r* = -0.67, *p* = 2.86e-12), implying an overall reduction in the correlation between ROIs. The subcortical covariance showed similar trends in comparison with those in cortical thickness (Fig. 2, Table S3).

We applied independent samples t-tests to estimate the sex differences in brain variability properties. Male and female participants showed significant differences after Bonferroni correction (*p* < 0.05/6 = 0.008) in cortical variance (*p* = 0.005, 95% confidence intervals (CI) [-0.059, -0.011]), cortical entropy (*p* = 9.82e-06, 95% CI [0.002, 0.005]), and subcortical variance (*p* = 0.003, 95% CI [-0.003, -0.001]), but not in the cortical proportion of pairs that differ significantly (*p* = 0.031, 95% CI [-0.036, -0.002]), subcortical entropy (*p* = 0.021, 95% CI [0.002, 0.030]), or the subcortical proportion of pairs that differ significantly (*p* = 0.001, 95% CI [-0.063, -0.015]). Cortical variances among females initially began lower than those among males in the younger age groups. However, with age, these variances increased gradually and eventually surpassed those of males around the age of 70. Similarly, a decrease in entropy and an increase in the proportion of pairs that differ significantly with increasing age were also observed among females (Fig. 2).

### Enrichment of correlation coefficients between and within lobar regions

In general, ROIs within the occipital lobe showed higher correlations across the all the age groups (for example, Fig. S1). This is also statistically significant as confirmed by the ORCA. The occipital lobe had significant enrichment of correlation coefficients above a threshold than by chance throughout all the ages (*p* < 0.05). Also, there were a greater number of significant negative correlations between the frontal and occipital lobes than expected (Fig. S1). ORCA showed that significant enrichment for higher correlations between occipital and frontal lobes was also quite stable across the age (Fig. 2B). More details can be found in Table S4.

### Test for equality of correlation coefficients between the youngest group versus other groups

**Fig. 3:**
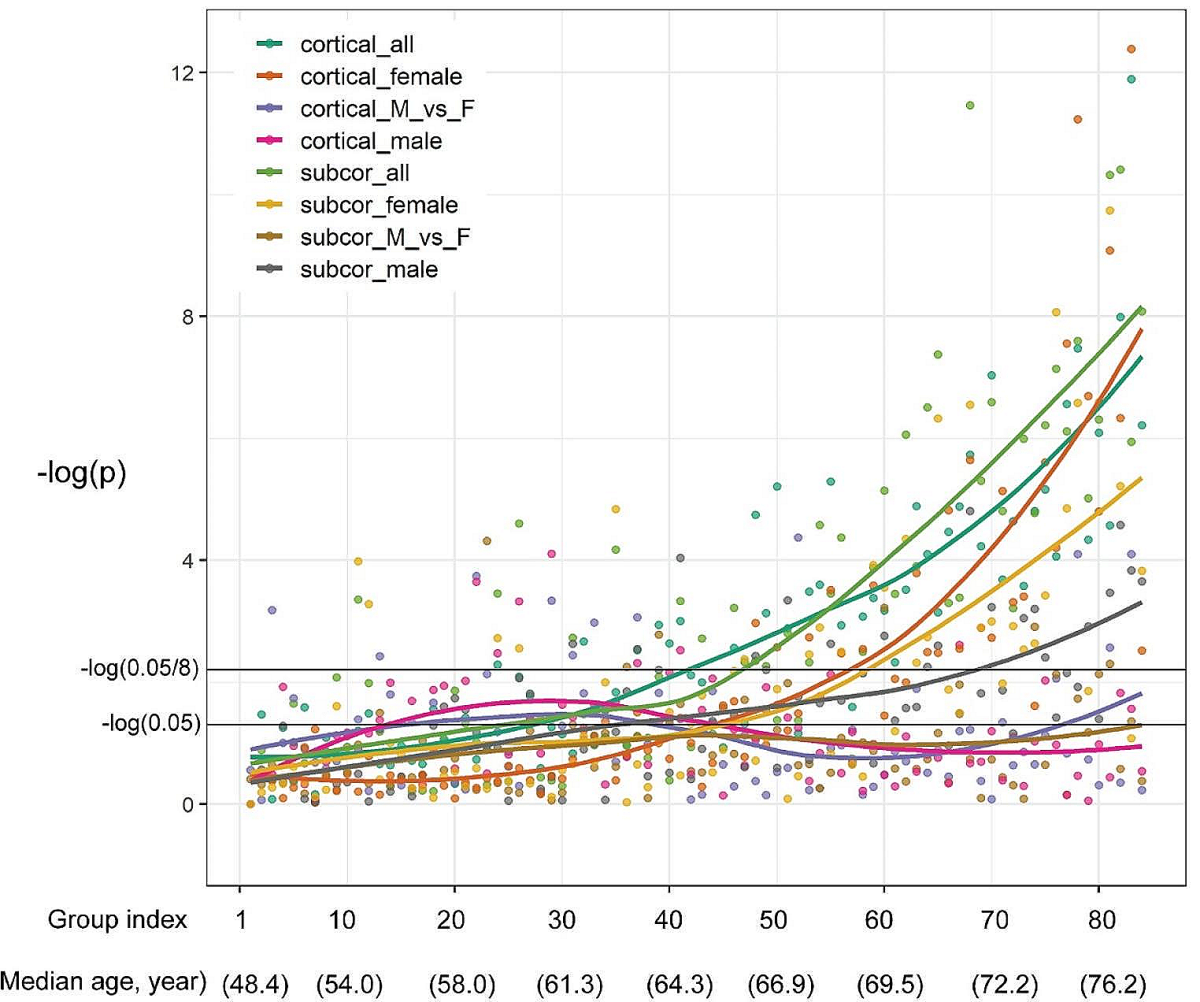
Test for equality of correlation coefficients between group 1 and each of other groups. The x-axis indicates group index (from 1 to 84) and corresponding median age of each group. The y-axis indicates − log_10_(p) for the test for equality of group 1 matrix and each of other matrices. The black horizontal line represents significant level (-log_10_[0.05]) and Bonferroni corrected p value (-log_10_[0.05/8]). The *cortical_all* represents the comparison of all participants’ correlation matrices between the first age group and each of other age groups (comparing group 1 with other groups). The *cortical_female* represents the comparison of only females’ correlation matrices between the first age group and each of other age groups (comparing group 1 with other groups). The *cortical_M_vs_F* represents the comparison of correlation matrices between males and females of the same age group (comparing males with females in each group). The *cortical_male* represents the comparison of only males’ correlation matrices between the first age group and each of other age groups (comparing group 1 with other groups). Other subcortical labels are similar to cortical labels

By comparing the elements of the correlation matrices in the first age group with corresponding elements of the correlation matrices of other age groups, we found that the correlation matrices of cortical thickness in older age were significantly different from that in the youngest group, especially after the age of around 64, with females showing more pronounced trends than males in both cortical thickness and subcortical volume matrices, which was indicated in Fig. 3, with a black horizontal line representing the statistically significant level. Additionally, by comparing the elements of the correlation matrices of males and females in each age group, we did not find any significant trends across all 84 age groups. More details can be found in Table S5.

### Associations between structural covariance and age

With 500 participants (the oldest age group has 575 participants) in each group, we calculated the median age and median cognition in each age group. The associations between structural covariance and these two measures (median age and median cognition) were estimated. The details of these median values in 84 groups can be found in Table S6.

#### Cortical thickness covariance

**Fig. 4:**
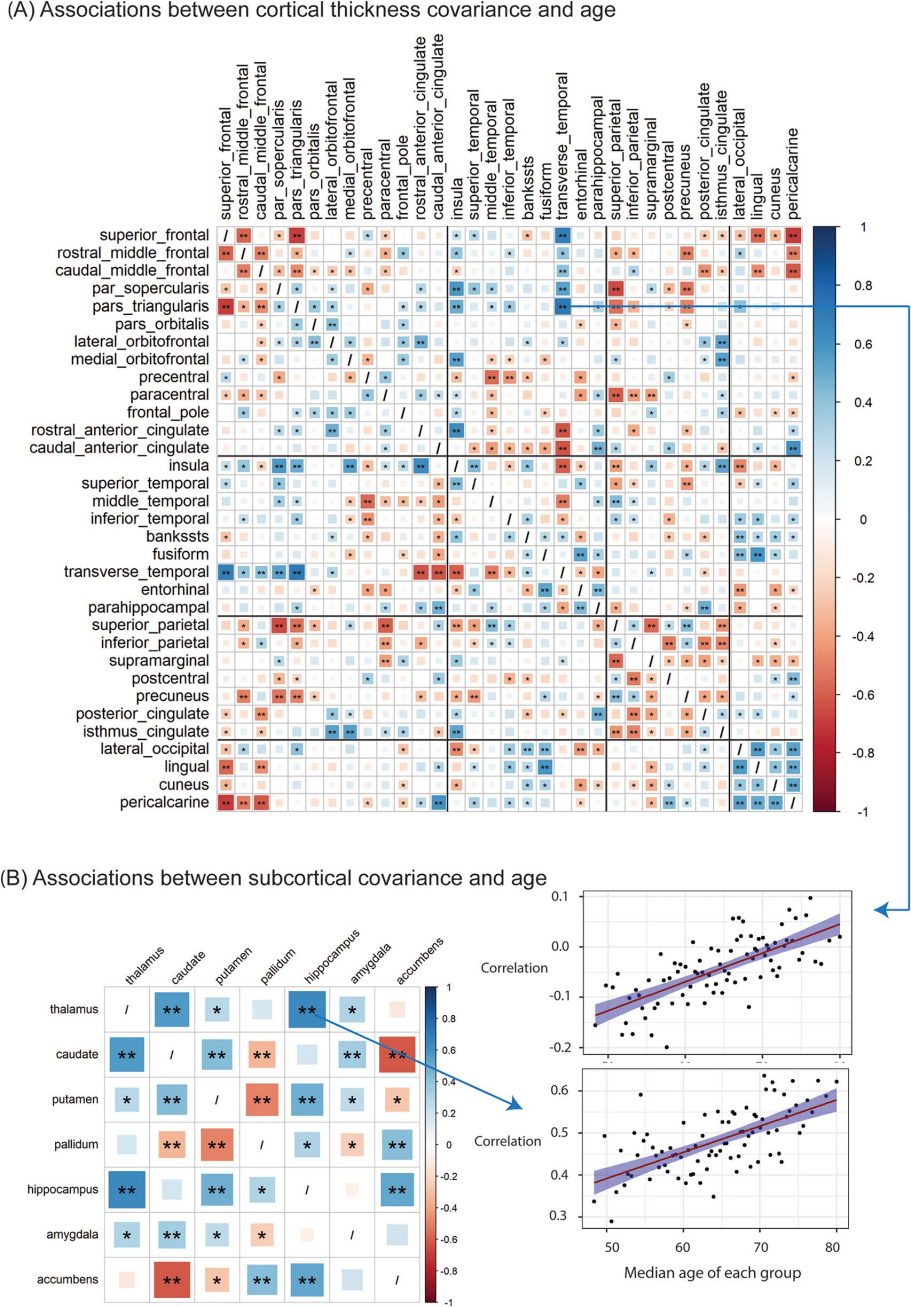
Associations between structural covariance and age in cortical thickness (**A**) and (**B**) subcortical volume. Every element in the matrix indicates the association between the pairwise correlation of brain structures and median age of each group. The single asterisk (*) represents the level of statistical significance *p* < 0.05. Double asterisks (**) represent the associations that remain statistically significant after Bonferroni correction

Each of the correlation coefficient across the age groups were tested for its association with median age. Sixty-two out of a total of 528 pairwise correlations were significantly associated with age across all 84 age groups after multiple test correction (Bonferroni correction, *p* < 0.05/528 = 9.47e-5) (Fig. 4A). With increasing age, some strongly negative correlations were observed between frontal lobe regions and other regions. The first four most significant pairwise correlations were between transverse temporal and pars triangularis (*r* = 0.71, *p* = 3.92e-14), pars triangularis and superior frontal (*r* = -0.69, *p* = 2.31e-13), pericalcarine and superior frontal (*r* = -0.69, *p* = 3.06e-13), and between transverse temporal and superior frontal (*r* = 0.69, *p* = 6.13e-13). The pairwise correlations between the regions within the occipital lobe all increased with age. All 62 significant associations can be found in Supplementary Fig. S2 and Table S7.

#### Subcortical covariance

Ten out of a total of 21 pairwise correlations were significantly associated with age across all 84 age groups after Bonferroni correction (*p* < 0.05/21 = 0.0024) (Fig. 4B). Notably, the correlations between hippocampus and other subcortical regions such as thalamus (*r* = 0.64, *p* = 3.57e-11), putamen (*r* = 0.49, *p* = 2.53e-06), and accumbens (*r* = 0.52, *p* = 4.43e-07) were significantly higher with increasing age. On the contrary, the correlation between caudate and accumbens was significantly decreased with increasing age (*r* = -0.62, *p* = 4.72e-10). Details of all 10 significant associations can be found in Supplementary Fig. S2 and Table S7.

### Additional analysis

Structural covariances in left and right hemispheres showed similar patterns to the covariance based on the average of the left and right hemispheres (Supplementary Fig. S3 and Fig. S4). Additionally, the analysis without removing global mean cortical thickness revealed predominantly positive pairwise correlations, particularly prominent in regions of the occipital lobe (Supplementary Fig. S5).

Through the application of different grouping methods, we discovered that the findings obtained from groups consisting of approximately 300 and 800 participants were similar to the results obtained from groups consisting of approximately 500 participants (Fig. S6 and Fig. S7).

The associations between pairwise correlations and global cognition were found to be largely opposite to their associations with age. This can be explained by the fact that cognition usually declines in the ageing process as shown in our data (Fig. S8). Associations between structural covariance and cognition can be found in Supplementary (Fig. S9 to Fig. S12).

## Discussion

We investigated age-related structural covariance properties and their associations with age and cognition using a large sample of 42,075 participants drawn from the UK Biobank. Firstly, with advancing age, there was a significant increase in the variance of whole brain structural covariance, indicating a greater variability in the relationships between brain regions. Additionally, we observed a decrease in entropy, suggesting a reduction in the complexity and diversity of these structural covariance patterns in older individuals. Secondly, there was significant and stable enrichment of pairwise correlations within the regions of the occipital lobe in the ageing process. Thirdly, both cortical thickness and subcortical volume covariances in the older groups were significantly different from those in the youngest group. This discrepancy was particularly pronounced among females. Fourthly, brain structural covariances were not stable though the ageing process, with the pairwise correlations between some brain regions strengthening and some weakening. Lastly, the significant pairwise correlations between brain regions were also associated with cognitive abilities.

Cortical integration means that different brain regions co-vary in pace or were shaped with similar biological processes (Nadig et al. 2021). Our findings suggest that brain structural covariance matrices have greater variability in older age, which may be speculated as cortical integration loosening in the older brain. Given the changes of structural covariance observed in the developmental stages during childhood and adolescence (Vijayakumar et al. 2021; Váša et al. 2018), we hypothesised that the structural covariance in the ageing brain would also be ‘dynamic’ rather than ‘static’; and our results confirmed this premise. One finding was that the variance of correlations within the matrix increased across 84 age groups, but the entropy decreased in the ageing brain. Similar phenomenon was found for Shannon entropy which is known to be negatively correlated with the variance (Wang et al. 2015). In the ageing brain, brain entropy quantifies brain’s capacity for adaptation, and smaller entropy corresponds to less complexity of the brain (Cieri et al. 2021). The decreased entropy in the ageing brain means the loss of complexity in brain connectivity networks, which may contribute to cognitive declines (Keshmiri 2020). Additionally, with ageing, females showed higher variance and lower entropy than males, suggesting potential differences in brain networks between genders. The observed sex differences on the whole brain variability properties may be attributed to known differences in brain structure and function between males and females (Canli et al. 2002; Cosgrove et al. 2007). However, our analysis did not specifically investigate their causal relationships, emphasizing the need for future studies designed to address this issue. Overall, our findings suggest that cortical integration appears to weaken in the ageing process.

By comparing covariance matrices of the youngest versus the older groups, we found that the older the age group was, the more significant the difference it had in comparison with the youngest group. There was a noticeable acceleration of the differences at ~ 64 years of age (age group #40), which suggests that age-related differences of human brain covariance begin at this age. This finding is in line with the findings of degenerative dementias, such as Alzheimer’s disease, whose rate of onset increases exponentially after the age of 65 (Fox and Schott 2004). It is worth noting that the structural covariances for females in older age diverged more from the youngest group than for males. This sex-related divergence was also evident in the differential rate of age-related increase of variance of whole brain covariances between males and females: females started lower than the males; they then surpassed the males at the age of 70 (Fig. 2).

The cortical thickness correlations between frontal lobe with other brain regions such as temporal, parietal, and occipital lobes were significantly different with ageing. For example, the correlations in superior and middle frontal-parietal, superior and middle frontal-occipital lobe were significantly decreased with advancing age. Notably, a positive correlation with age could signify either a strengthening of a pairwise correlation or a weakening of an anti-correlation. Similarly, a negative age correlation can imply either weakening or strengthening, depending on the direction and strength of the correlation. Associations between the anterior cingulate and transverse temporal regions are good examples to demonstrate this (Fig. S2, pair index 305 and 325). The existence of both positive and negative correlations in relation to older age between certain brain regions suggests that the interaction between these brain regions was variable: some correlations were strengthening, some weakening, and some were reversed in direction over time. Given that brain structures generally decline in the ageing brain, such as cortical thinning and subcortical atrophy, our finding demonstrates that there are diverse ageing-related differences occurring in different brain structures. We found that the regions in the occipital lobe were highly correlated with each other, and it has been reported that they share similar genetic underpinnings (Grasby et al. 2020; Hofer et al. 2020). This could also perhaps explain our observed enrichment of correlation coefficients within the occipital lobe. Our findings indicate that the enrichment in occipital correlations was significant though the ageing process, and these correlations were significantly associated with age as well. Additionally, our findings indicated that when mean cortical thickness was not controlled for, most pairwise correlations were positive and showed an increase across age groups, unlike the results when controlling for mean cortical thickness. This suggests that mean cortical thickness influences pairwise correlations, particularly in older age groups, where regions tend to be more closely associated.

Like cortical thickness, subcortical volumetric covariances were also associated with the ageing process. As one of the important subcortical regions, the hippocampus plays a critical role in memory and learning, as well as spatial navigation (Burgess et al. 2002). It is vulnerable to neurodegenerative diseases, especially Alzheimer’s disease (Mu and Gage 2011). In our analysis, the correlations between hippocampal volume with thalamus, putamen, and accumbens, were all significantly increased with increasing age, suggesting synchronised patterns in their volumes during the ageing process. The significantly decreased correlations between accumbens and caudate, putamen and pallidum, caudate and pallidum may indicate that these pairs of subcortical structures have independent trajectories during the ageing process. The amygdala and hippocampus are key components of the medial temporal lobe and are involved in emotional perception and regulation (Groen et al. 2010). Our study showed that the covariance between these two regions remained relatively stable during ageing.

We found significant associations between structural covariance and global cognition, processing speed, executive function, as well as memory. Previous work has shown that structural covariance of the default network was associated with cognitive ability (Spreng and Turner 2013), and that the synergy in the human brain may have evolved to support higher cognitive function (Luppi et al. 2022). Additionally, our finding of a high correlation between cognition and age (Fig. S8) was not on the individual level. Instead, the correlations were calculated by using the median age and median cognition of each group across all 84 age groups, thus the individual variance of each group for these measures was effectively smoothed out. Given the highly negative correlation between the age and cognition, as well as the associations between the structural covariance and age found in our study, it is not surprising that there were also associations between structural covariance and cognition, and their association direction was opposite to that of between structural covariance and age. In contrast to the positive associations between subcortical covariances and age, many subcortical covariances were found to be negatively associated with global cognition across all 84 age groups, especially the correlations between hippocampus and thalamus and those between caudate and thalamus.

Our study has some limitations. First, our data were cross-sectional in nature, which would not allow causal interpretation for the relationships of structural covariances and their relationships with cognition. Our study design which divided participants into 84 age groups allowed us to explore age related differences in structural covariance, but these differences should not be interpreted as real changes. Second, in order to explore the associations between structural covariance and age, we computed brain structural covariance in the group with 500 participants. This grouping was empirically explored and decided, and there may be biases associated with such grouping. We, therefore, also tested grouping of 300 and 800 participants. Robustness for each group increased with the increase of group size, but increasing group size would result in smaller number of groups. While there is no fixed rule for this, we did consider several factors, such as statistical power, stability and reliability of the correlations, the study design, and our research question. Third, we acknowledge the potential impact of noise on our findings, which may stem from FreeSurfer estimation error. Additionally, due to ageing, older populations may have greater variances in cortical thickness/volumetric data compared to younger (middle-aged) individuals. Finally, the generalisability of our results may not extend to other racial/ethnic groups, as we restricted our analyses to individuals of British ancestry.

In conclusion, we utilized a large cross-sectional dataset to provide an overview of associations between structural covariance and age, revealing varying regional interactions related to brain morphology in ageing. These findings could help better understand how brain regions interplay with each other during the ageing process.

### Electronic supplementary material

Below is the link to the electronic supplementary material.

Supplementary Material 1

Supplementary Material 2

## Data Availability

Data for UK Biobank can be accessed by formal application (https://www.ukbiobank.ac.uk/). The code used during the current study is available from the corresponding author on reasonable request.

## References

[CR1] Alexander-Bloch A, Giedd JN, Bullmore E (2013). Imaging structural co-variance between human brain regions. Nat Rev Neurosci.

[CR2] Bishop NA, Lu T, Yankner BA (2010). Neural mechanisms of ageing and cognitive decline. Nature.

[CR3] Burgess N, Maguire EA, O’Keefe J (2002). The human hippocampus and spatial and episodic memory. Neuron.

[CR4] Canli T, Desmond JE, Zhao Z, Gabrieli JDE (2002) Sex differences in the neural basis of emotional memories. Proceedings of the National Academy of Sciences 99 (16):10789–10794. 10.1073/pnas.162356599 10.1073/pnas.162356599 PMC125046 12145327

[CR5] Carmon J, Heege J, Necus JH, Owen TW (2020). Reliability and comparability of human brain structural covariance networks. NeuroImage.

[CR6] Cieri F, Zhuang X, Caldwell JZK, Cordes D (2021) Brain Entropy during Aging through a Free Energy Principle Approach. Front Hum Neurosci 15. 10.3389/fnhum.2021.647513 10.3389/fnhum.2021.647513 PMC8019811 33828471

[CR7] Cosgrove KP, Mazure CM, Staley JK (2007). Evolving knowledge of sex differences in brain structure, function, and chemistry. Biol Psychiatry.

[CR8] de Jong LW, van der Hiele K, Veer IM, Houwing JJ (2008). Strongly reduced volumes of putamen and thalamus in Alzheimer’s disease: an MRI study. Brain.

[CR9] Desikan RS, Ségonne F, Fischl B, Quinn BT (2006). An automated labeling system for subdividing the human cerebral cortex on MRI scans into gyral based regions of interest. NeuroImage.

[CR10] Diedenhofen B, Musch J (2015). Cocor: a comprehensive solution for the statistical comparison of correlations. PLoS ONE.

[CR11] Du J, Koch FC, Xia A, Jiang J (2021). Difference in distribution functions: a new diffusion weighted imaging metric for estimating white matter integrity. NeuroImage.

[CR12] DuPre E, Spreng RN (2017). Structural covariance networks across the life span, from 6 to 94 years of age. Netw Neurosci.

[CR13] Felippe HS, Viol A, Araujo, DBd, Luz, MGEd et al (2021) The Von Neumann entropy for the Pearson correlation matrix: a test of the entropic brain hypothesis. 10.48550/arXiv.2106.05379. arXiv:2106.05379

[CR14] Fischl B (2012). FreeSurfer Neuroimage.

[CR15] Fjell AM, Westlye LT, Amlien I, Espeseth T (2009). High consistency of Regional cortical thinning in aging across multiple samples. Cereb Cortex.

[CR16] Fouladi RT, Serafini PE (2018) MML-WBCORR. Retrieved from https://shiny.rcg.sfu.ca/u/zrauf/MML-WBCORR/

[CR17] Fox NC, Schott JM (2004). Imaging cerebral atrophy: normal ageing to Alzheimer’s disease. Lancet.

[CR18] Grady C (2012). The cognitive neuroscience of ageing. Nat Rev Neurosci.

[CR19] Grasby KL, Jahanshad N, Painter JN, Colodro-Conde L (2020). The genetic architecture of the human cerebral cortex. Science.

[CR20] Groen W, Teluij M, Buitelaar J, Tendolkar I (2010). Amygdala and hippocampus enlargement during adolescence in autism. J Am Acad Child Adolesc Psychiatry.

[CR21] Hafkemeijer A, Altmann-Schneider I, de Craen AJM, Slagboom PE (2014). Associations between age and gray matter volume in anatomical brain networks in middle-aged to older adults. Aging Cell.

[CR22] Hofer E, Roshchupkin GV, Adams HHH, Knol MJ (2020). Genetic correlations and genome-wide associations of cortical structure in general population samples of 22,824 adults. Nat Commun.

[CR23] Jia Y, Gu H, Luo Q (2017). Sample entropy reveals an age-related reduction in the complexity of dynamic brain. Sci Rep.

[CR24] Keshmiri S (2020). Entropy and the brain: an overview. Entropy.

[CR25] Luppi AI, Mediano PAM, Rosas FE, Holland N (2022). A synergistic core for human brain evolution and cognition. Nat Neurosci.

[CR26] Mechelli A, Friston KJ, Frackowiak RS, Price CJ (2005). Structural covariance in the human cortex. J Neuroscience: Official J Soc Neurosci.

[CR27] Montembeault M, Joubert S, Doyon J, Carrier J (2012). The impact of aging on gray matter structural covariance networks. NeuroImage.

[CR28] Montembeault M, Rouleau I, Provost J-S, Brambati SM (2016). Altered Gray Matter Structural Covariance Networks in Early stages of Alzheimer’s Disease. Cereb Cortex.

[CR29] Mu Y, Gage FH (2011). Adult hippocampal neurogenesis and its role in Alzheimer’s disease. Mol Neurodegeneration.

[CR30] Nadig A, Seidlitz J, McDermott CL, Liu S et al (2021) Morphological integration of the human brain across adolescence and adulthood. Proceedings of the National Academy of Sciences 118 (14). 10.1073/pnas.2023860118 10.1073/pnas.2023860118 PMC8040585 33811142

[CR31] Nestor SM, Mišić B, Ramirez J, Zhao J (2017). Small vessel disease is linked to disrupted structural network covariance in Alzheimer’s disease. Alzheimer’s Dement.

[CR32] Pomyen Y, Segura M, Ebbels TMD, Keun HC (2015). Over-representation of correlation analysis (ORCA): a method for identifying associations between variable sets. Bioinformatics.

[CR33] Shannon CE (1948). A mathematical theory of communication. Bell Syst Tech J.

[CR34] Sotiras A, Toledo JB, Gur RE, Gur RC et al (2017) Patterns of coordinated cortical remodeling during adolescence and their associations with functional specialization and evolutionary expansion. Proceedings of the National Academy of Sciences 114 (13):3527–3532. 10.1073/pnas.1620928114 10.1073/pnas.1620928114 PMC5380071 28289224

[CR35] Spreng RN, Gary RT (2013). Structural covariance of the Default Network in healthy and pathological aging. J Neurosci.

[CR36] Spreng RN, Turner GR (2013). Structural covariance of the Default Network in healthy and pathological aging. J Neurosci.

[CR37] Steiger JHJP (1980). Tests for comparing elements of a correlation matrix. Psychol Bull.

[CR38] Sudlow C, Gallacher J, Allen N, Beral V (2015). UK biobank: an open access resource for identifying the causes of a wide range of complex diseases of middle and old age. PLoS Med.

[CR39] Tinaz S, Courtney MG, Stern CE (2011). Focal cortical and subcortical atrophy in early Parkinson’s disease. Mov Disord.

[CR40] Váša F, Seidlitz J, Romero-Garcia R, Whitaker KJ (2018). Adolescent Tuning of Association Cortex in Human Structural Brain Networks. Cereb Cortex.

[CR41] Vijayakumar N, Ball G, Seal ML, Mundy L (2021). The development of structural covariance networks during the transition from childhood to adolescence. Sci Rep.

[CR42] Wang K, Phillips CA, Saxton AM, Langston MA (2015). EntropyExplorer: an R package for computing and comparing differential Shannon Entropy, differential coefficient of variation and differential expression. BMC Res Notes.

[CR43] Zielinski BA, Gennatas ED, Zhou J, Seeley WW (2010) Network-level structural covariance in the developing brain. Proceedings of the National Academy of Sciences 107 (42):18191–18196. 10.1073/pnas.1003109107 10.1073/pnas.1003109107 PMC2964249 20921389

