# Supplementary material for: Brain structural covariances in the ageing brain in the UK Biobank
Source: Brain Struct Funct. 2024 Apr 16;229(5):1165–77. doi: 10.1007/s00429-024-02794-4 (PMC11147885; doi:10.1007/s00429-024-02794-4)
Supplement: Supplementary file 1 — Supplementary Material 1 [file 429_2024_2794_MOESM1_ESM.docx]

**Contents**

[Fig. S1. The cortical thickness covariance (correlation matrix) in group 1, 2, 83, and 84. 2](#_Toc162434356)

[Fig. S2. Significant associations between pairwise correlation and age in cortical thickness (62 pairs) and subcortical volume (10 pairs). 3](#_Toc162434357)

[Fig. S3. Associations between left hemisphere structural covariance and age 7](#_Toc162434358)

[Fig. S4. Associations between right hemisphere structural covariance and age 8](#_Toc162434359)

[Fig. S5. Associations between structural covariance and age without removing global mean cortical thickness. 9](#_Toc162434360)

[Fig. S6. Associations between structural covariance and age with 300 participants in each group 10](#_Toc162434361)

[Fig. S7. Associations between structural covariance and age with 800 participants in each group 11](#_Toc162434362)

[Fig. S8. The association between global cognition and age across 84 age groups. 12](#_Toc162434363)

[Fig. S9. Associations between structural covariance and age/global cognition. 13](#_Toc162434364)

[Fig. S10. Associations between structural covariance and age/processing speed. 14](#_Toc162434365)

[Fig. S11. Associations between structural covariance and age/executive function. 15](#_Toc162434366)

[Fig. S12. Associations between structural covariance and age/memory. 16](#_Toc162434367)

### Fig. S1. The cortical thickness covariance (correlation matrix) in group 1, 2, 83, and 84.


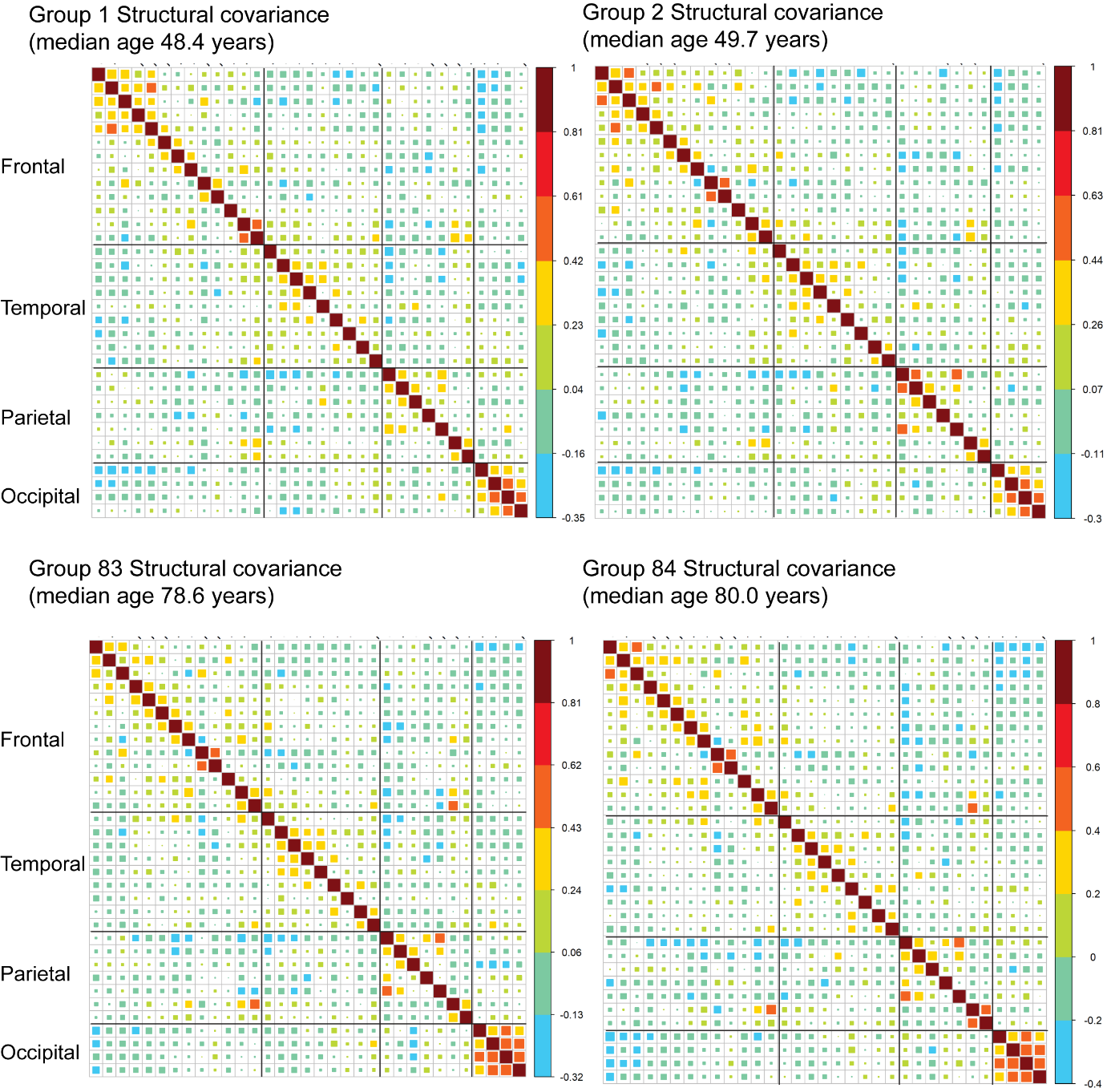


**Fig. S1**. The cortical thickness covariance (correlation matrix) in group 1, 2, 83, and 84. The elements of each correlation matrix represent cortical thickness correlations between any two brain regions regressing out sex, scanner, and global mean cortical thickness.

### Fig. S2. Significant associations between pairwise correlation and age in cortical thickness (62 pairs) and subcortical volume (10 pairs).


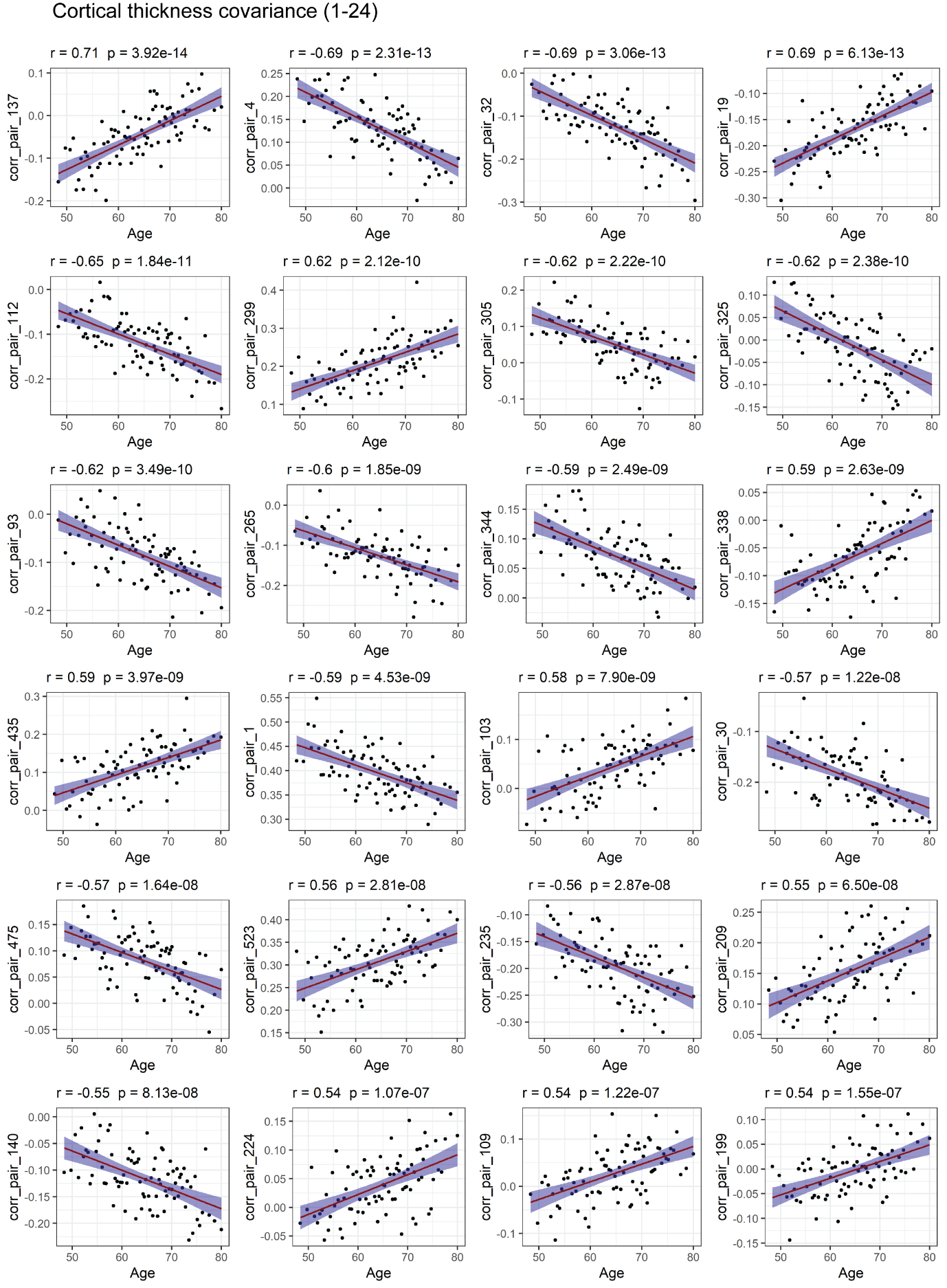


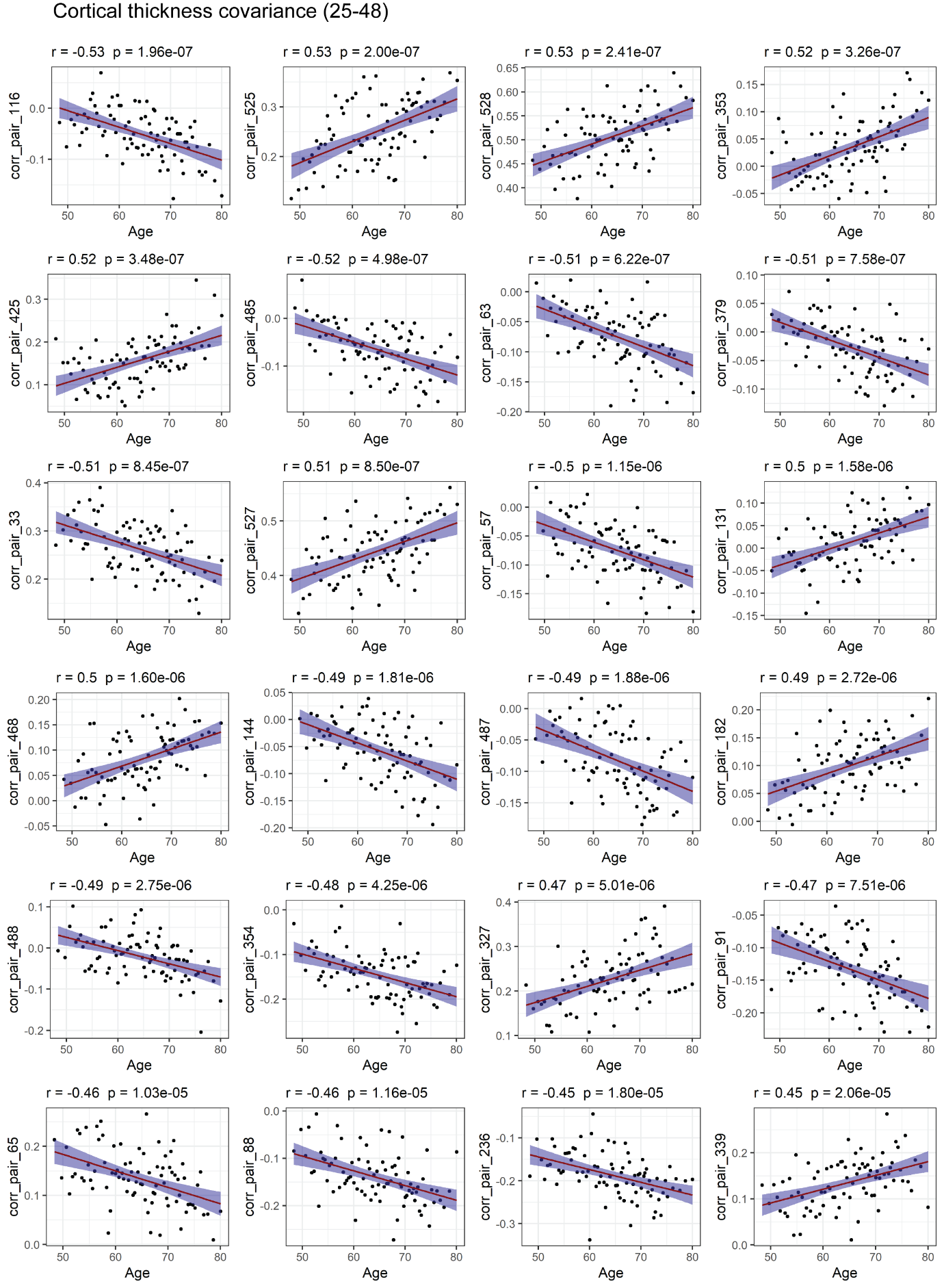


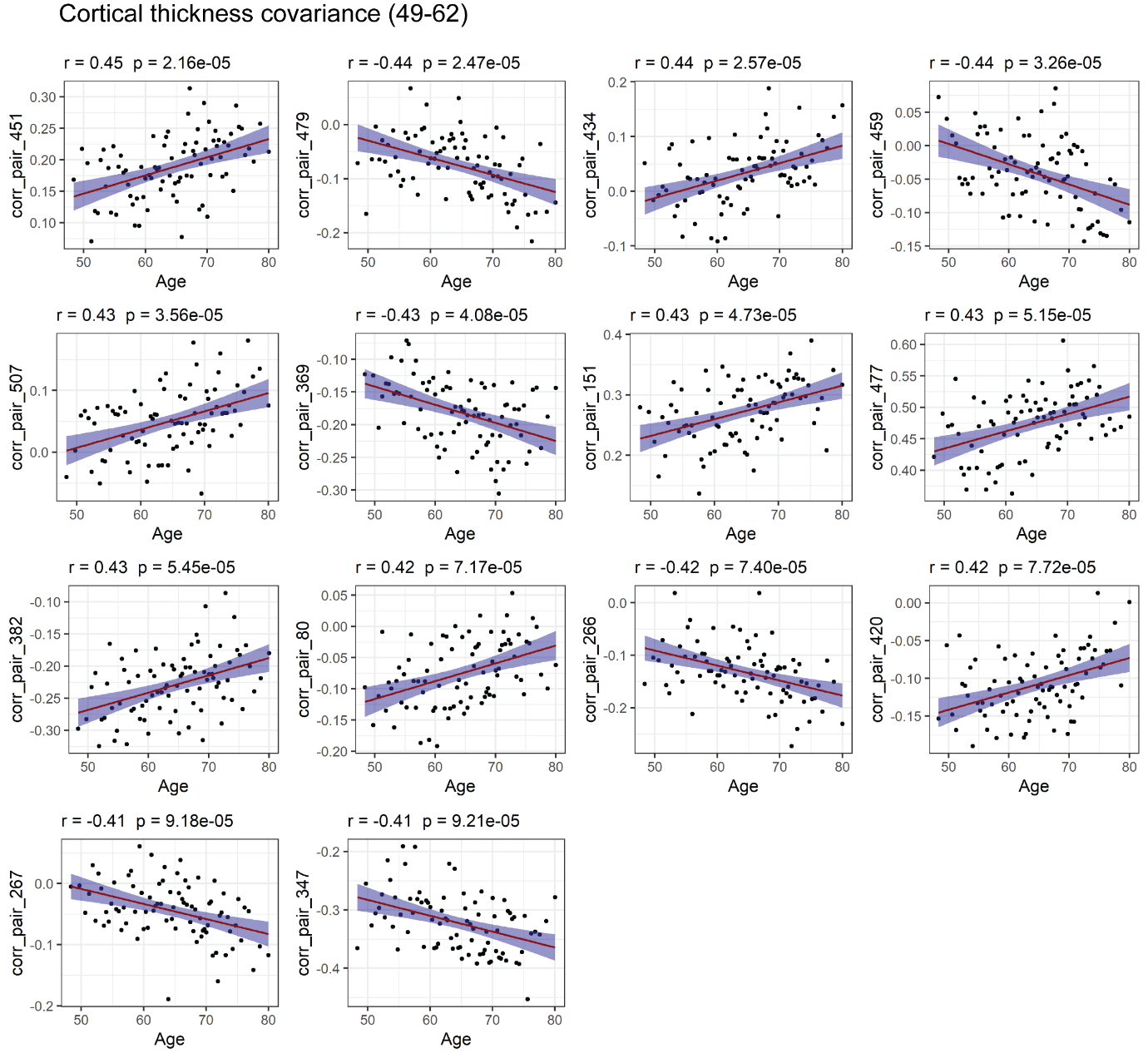


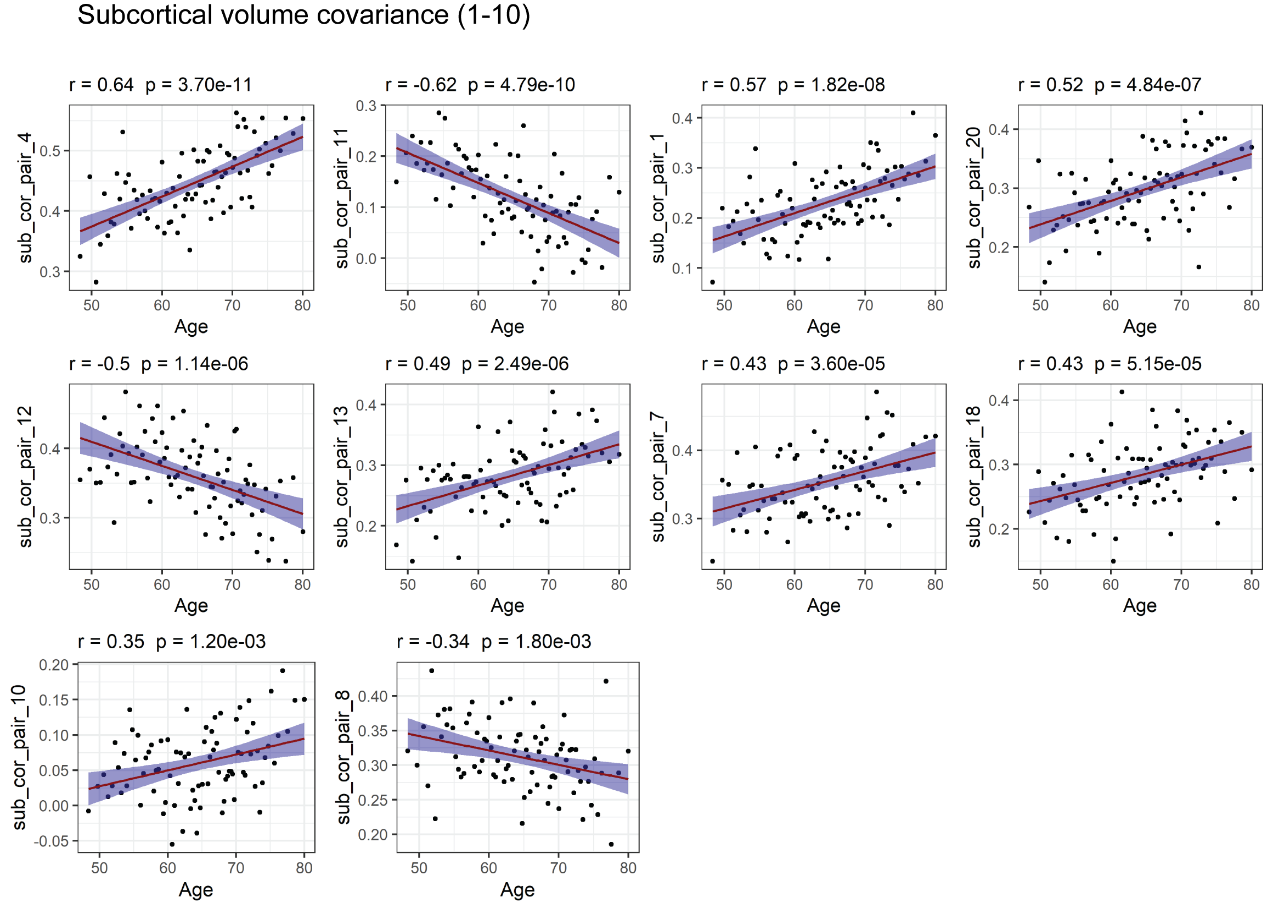


**Fig. S2**. All 62 pairs of significant associations between cortical pairwise correlation and age in cortical thickness and 10 pairs in subcortical volume. The x axis indicates median age in each age group. The y axis indicates the cortical pairwise correlation, and corresponding index of pairwise correlation can be found in Table S1.

### Fig. S3. Associations between left hemisphere structural covariance and age


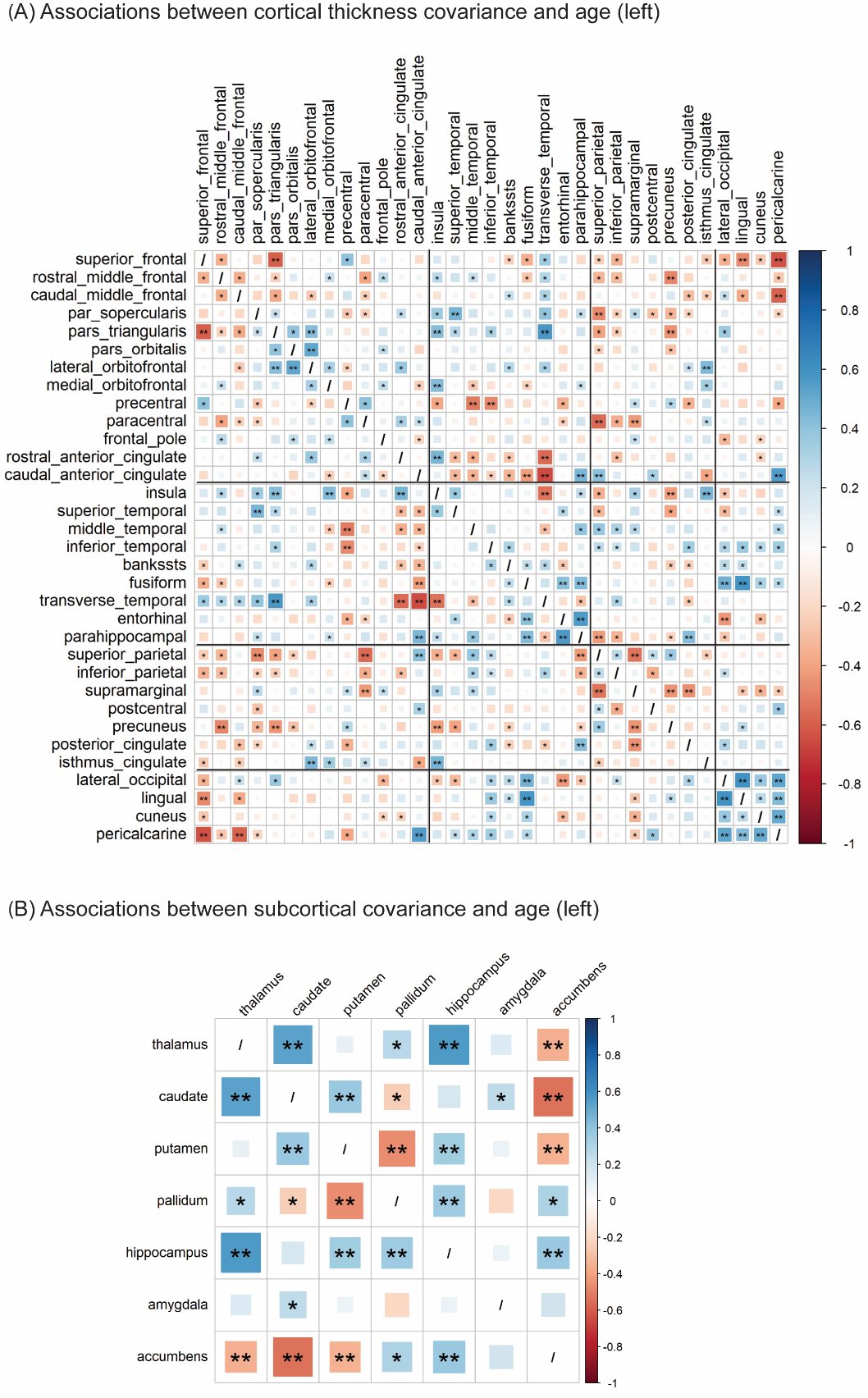


**Fig. S3**. Associations between left hemisphere structural covariance and age in cortical thickness (A) and (B) subcortical volume. Every element in the matrix indicates the association between the pairwise correlation of brain structures and median age in each group. The single asterisk (*) represents the level of statistical significance p<0.05. Double asterisks (**) represent the associations that remain statistically significant after Bonferroni correction.

### Fig. S4. Associations between right hemisphere structural covariance and age


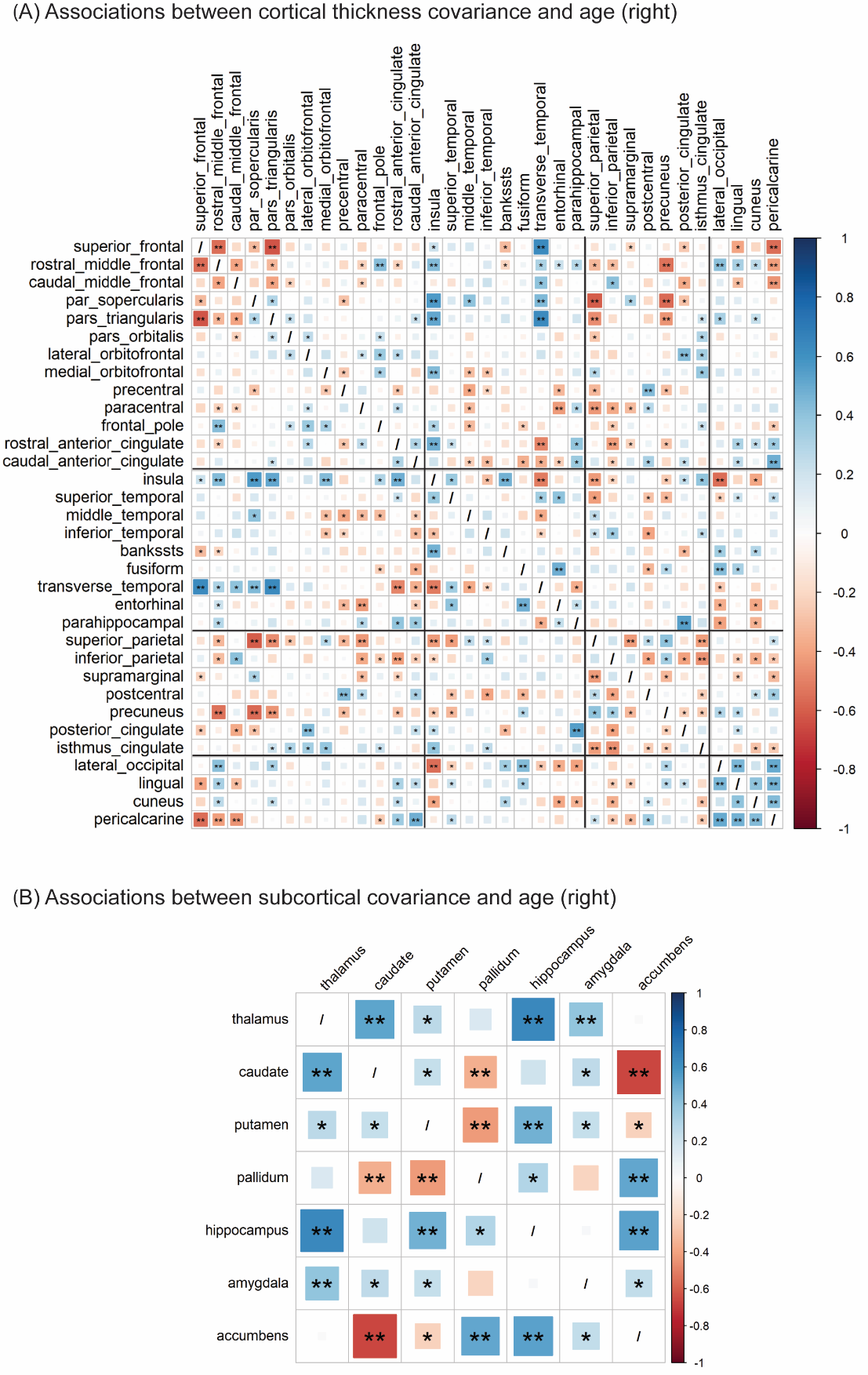


**Fig. S4**. Associations between right hemisphere structural covariance and age in cortical thickness (A) and (B) subcortical volume. Every element in the matrix indicates the association between the pairwise correlation of brain structures and median age in each group. The single asterisk (*) represents the level of statistical significance p<0.05. Double asterisks (**) represent the associations that remain statistically significant after Bonferroni correction.

### Fig. S5. Associations between structural covariance and age without removing global mean cortical thickness.


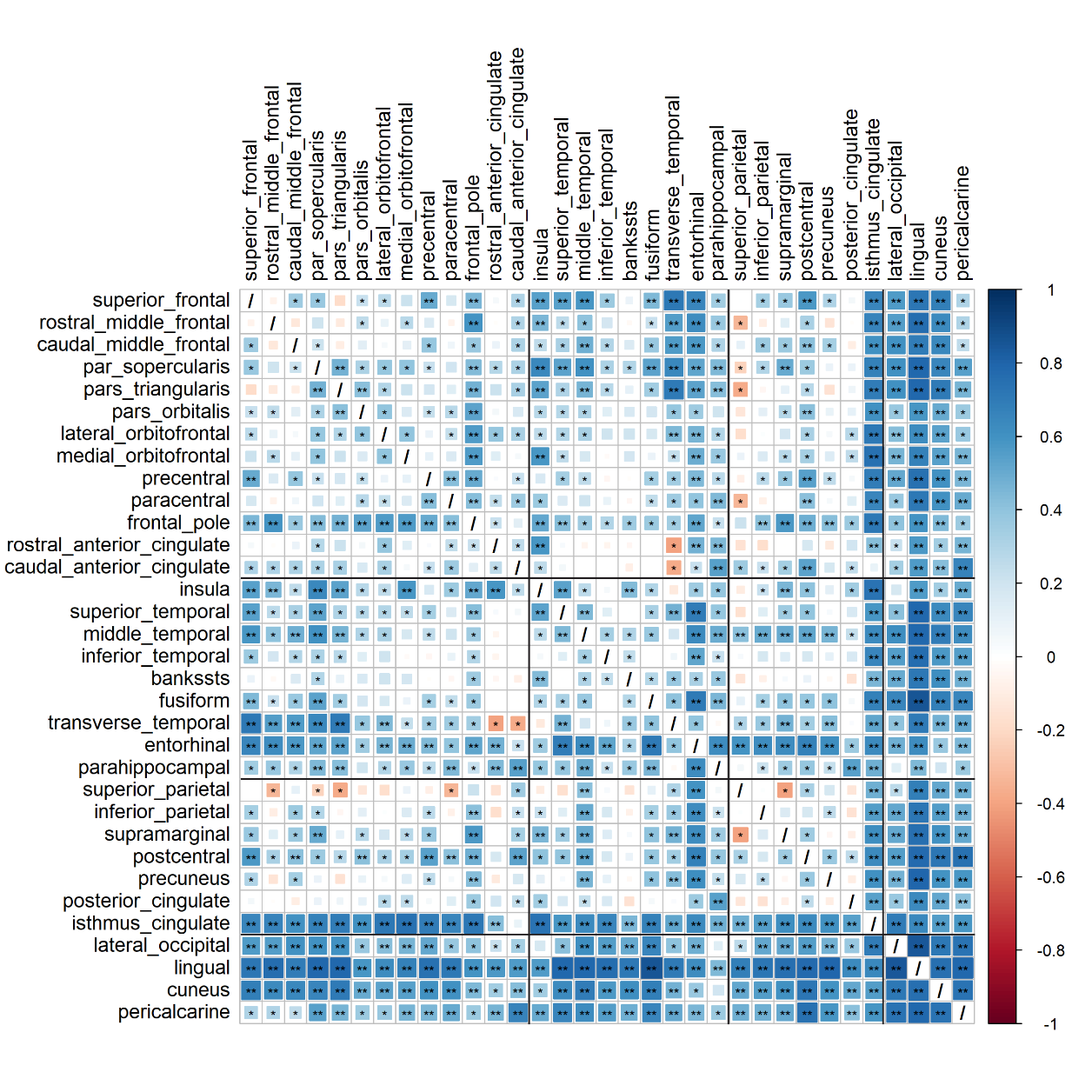


**Fig. S5**. Associations between structural covariance and age without removing global mean cortical thickness. Every element in the matrix indicates the association between the pairwise correlation of brain structures and median age in each group. The single asterisk (*) represents the level of statistical significance p<0.05. Double asterisks (**) represent the associations that remain statistically significant after Bonferroni correction.

### Fig. S6. Associations between structural covariance and age with 300 participants in each group


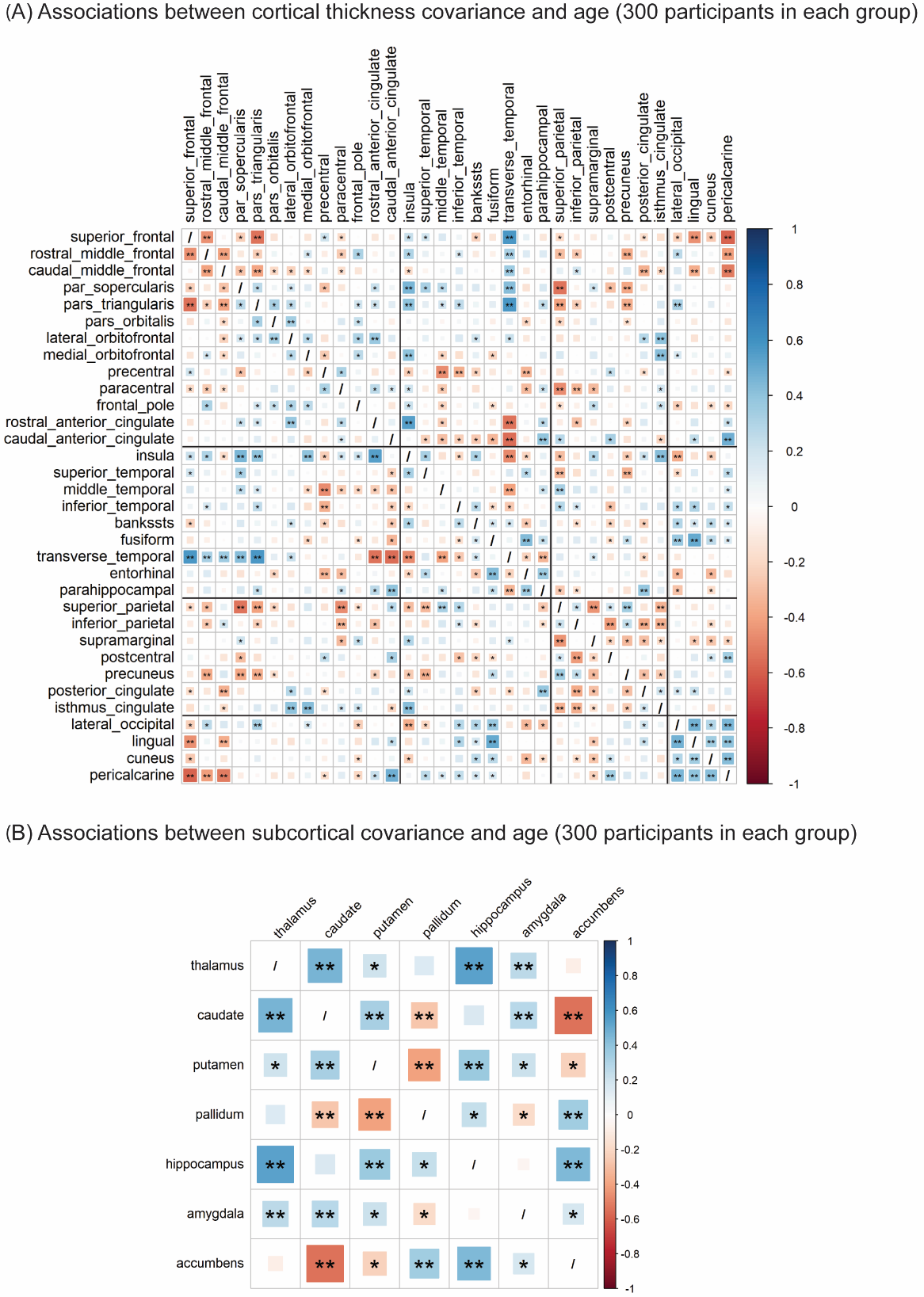


**Fig. S6**. Associations between structural covariance and age with 300 participants in each group.

### Fig. S7. Associations between structural covariance and age with 800 participants in each group


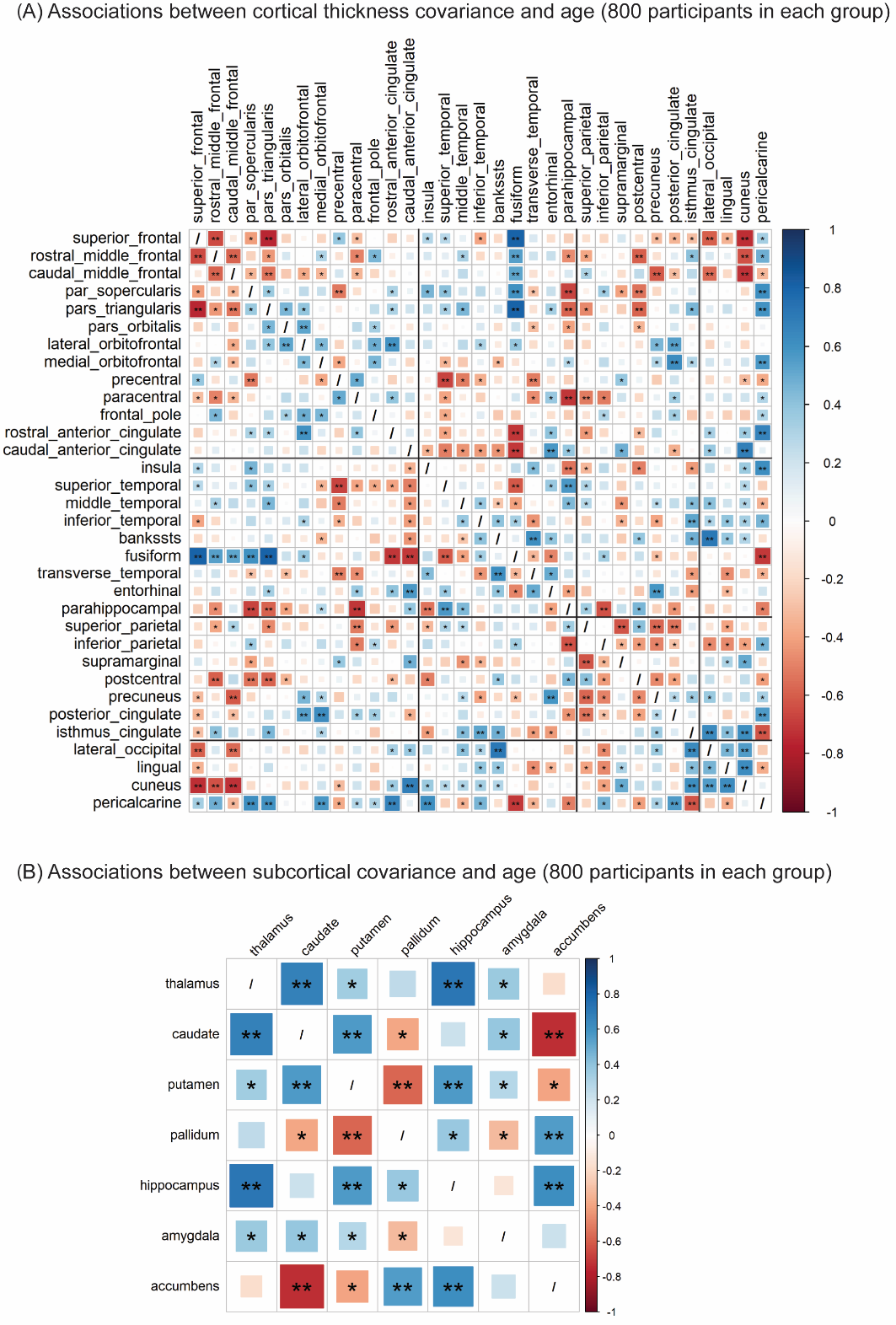


**Fig. S7**. Associations between structural covariance and age with 800 participants in each group.

**Associations between structural covariance and cognition**

Cortical thickness covariance: The associations between pairwise correlations and global cognition were found to be largely opposite to their associations with age, i.e., greater pairwise correlations were associated with both older age and poorer cognitive performance. This can be explained by the fact that cognition usually declines in the ageing process as shown in our data (Fig. S8). In addition, all the pairwise correlations within occipital lobe were significantly increased with older age and worse cognition (Fig. S9). The associations between covariance and specific cognitive domains (processing speed, executive function, and memory) were shown in Fig S10-S12. They all had similar association patterns.

Subcortical covariance: Similar findings were seen in subcortical covariance (Fig. S9). For example, the correlation between caudate and thalamus was positively associated with age (r = 0.57, p = 1.89e-08) but negatively associated with global cognition (r = -0.59, p = 2.57e-09). The associations between subcortical covariance and specific cognitive domains can be found in Fig. S10 to Fig. S12.

Further details about associations between pairwise brain region correlations and global cognition can be found in Table S8.

### Fig. S8. The association between global cognition and age across 84 age groups.


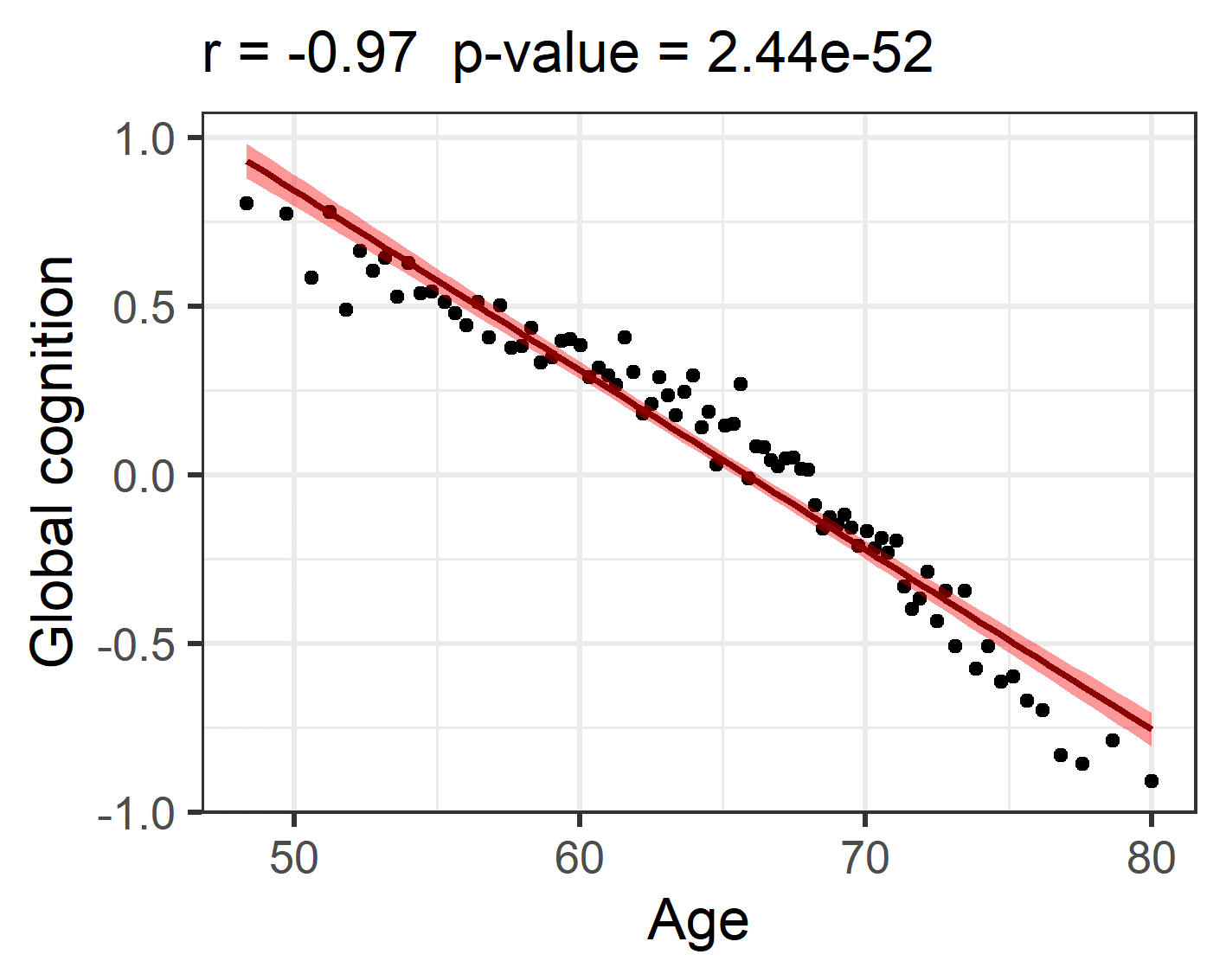


**Fig. S8**. The association between global cognition (z-transformed) and age across 84 age groups. Age and global cognition represent the median values in each age group.

### Fig. S9. Associations between structural covariance and age/global cognition.


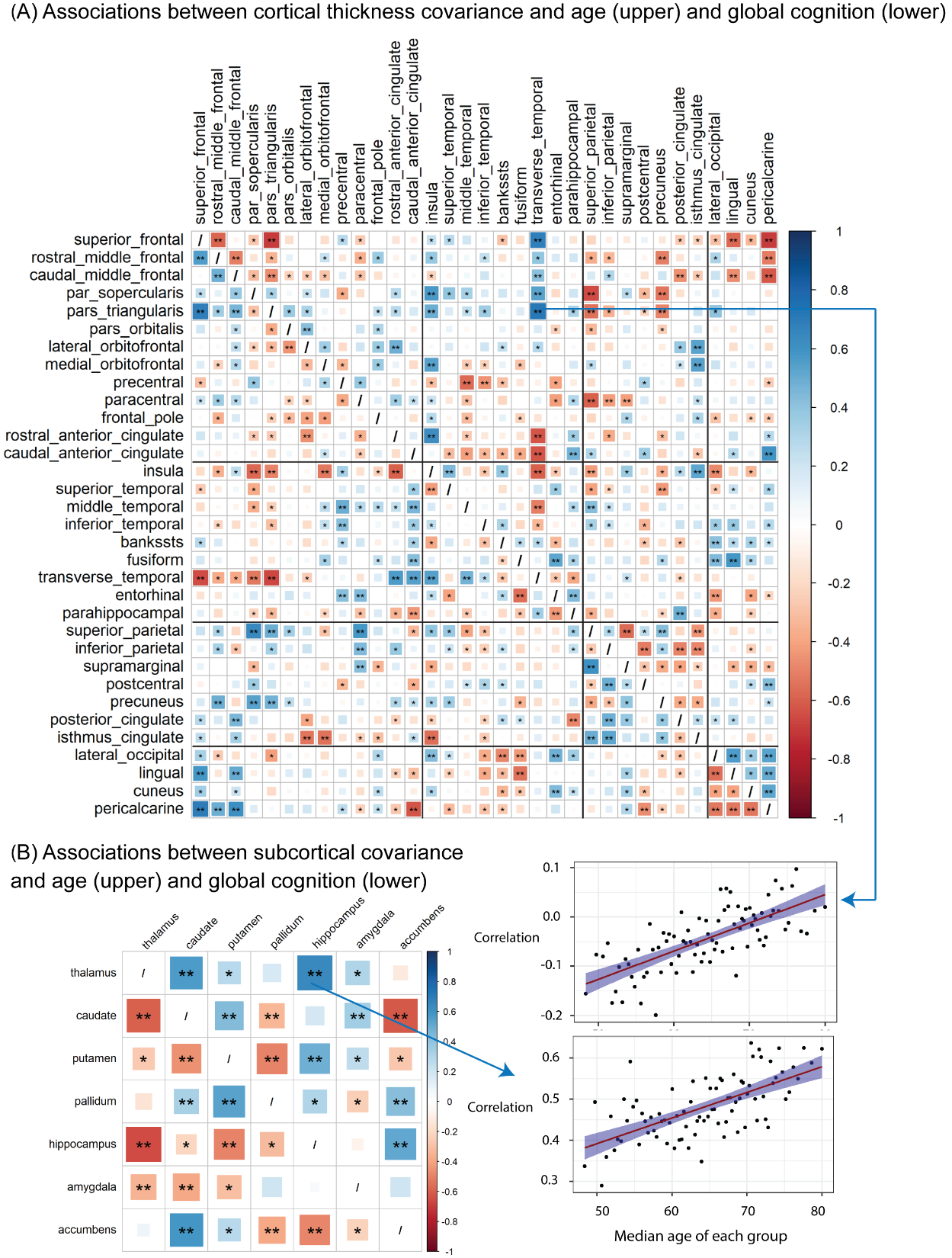


**Fig. S9**. Associations between structural covariance and age/global cognition in cortical thickness (A) and (B) subcortical volume. Covariance-age associations are shown in the upper right triangle and covariance- global cognition associations are shown in the lower left triangle. In the upper right triangle, every element in the matrix indicates the association between the pairwise correlation of brain structures and median age in each group. In the lower left triangle, every element in the matrix indicates an association between pairwise correlation of brain structures and global cognition. The single asterisk (*) represents the level of statistical significance p<0.05. Double asterisks (**) represent the associations that remain statistically significant after Bonferroni correction.

### Fig. S10. Associations between structural covariance and age/processing speed.


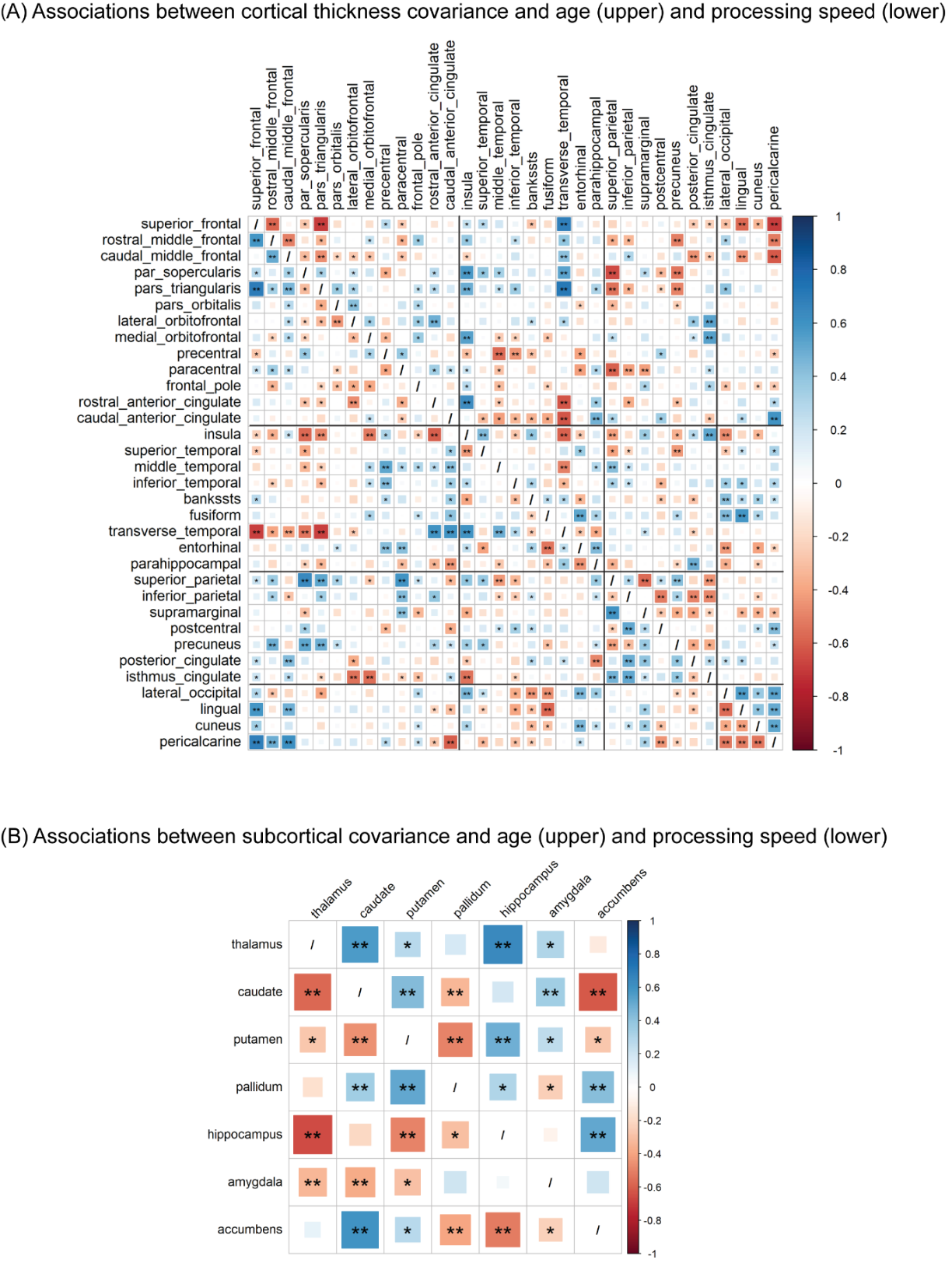


**Fig. S10**. Associations between structural covariance and age/processing speed in cortical thickness (A) and (B) subcortical volume. Covariance-age associations are shown in the upper right triangle and covariance-processing speed associations are shown in the lower left triangle.

### Fig. S11. Associations between structural covariance and age/executive function.


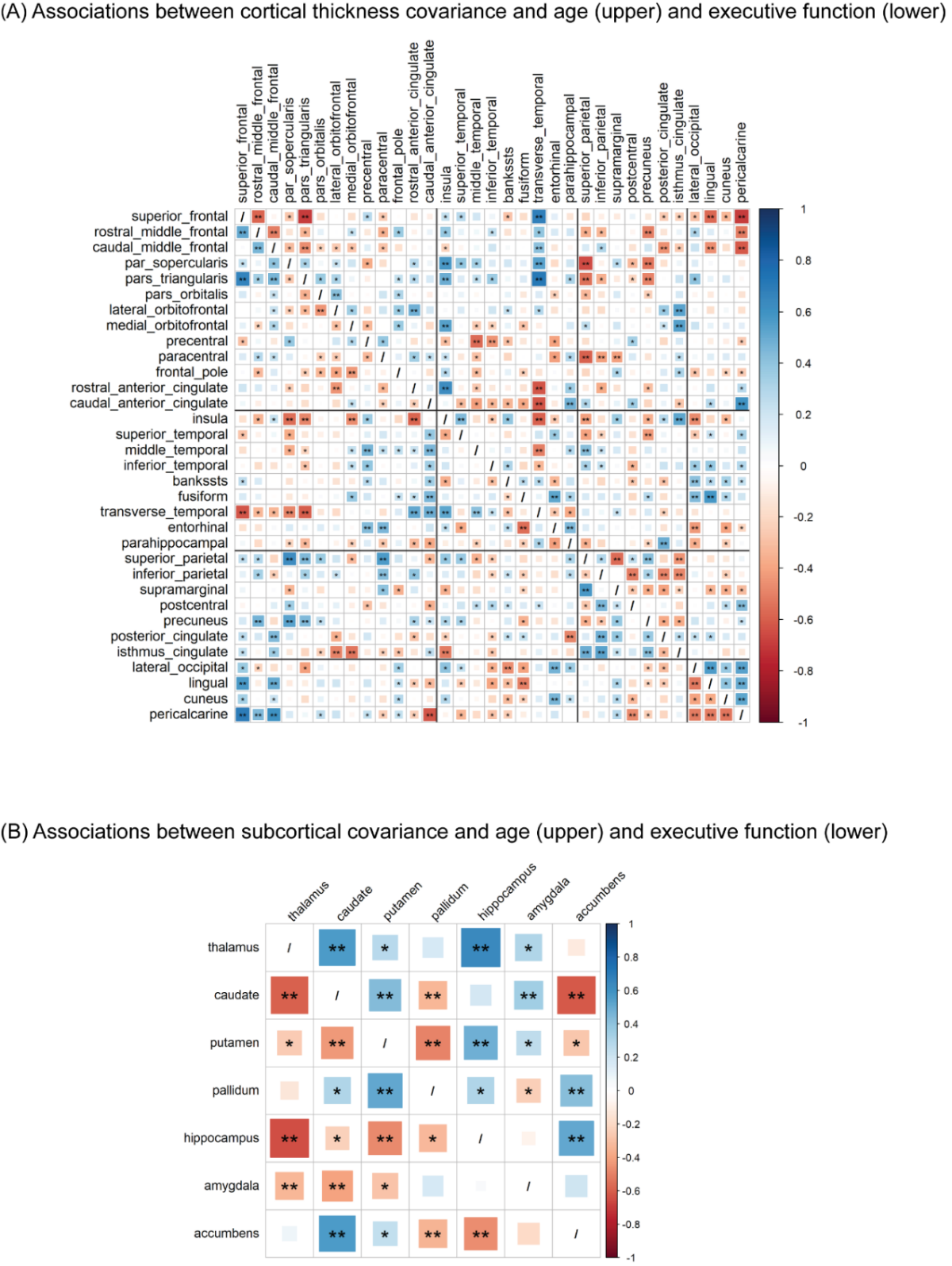


**Fig. S11**. Associations between structural covariance and age/executive function in cortical thickness (A) and (B) subcortical volume. Covariance-age associations are shown in the upper right triangle and covariance-executive function associations are shown in the lower left triangle.

### Fig. S12. Associations between structural covariance and age/memory.


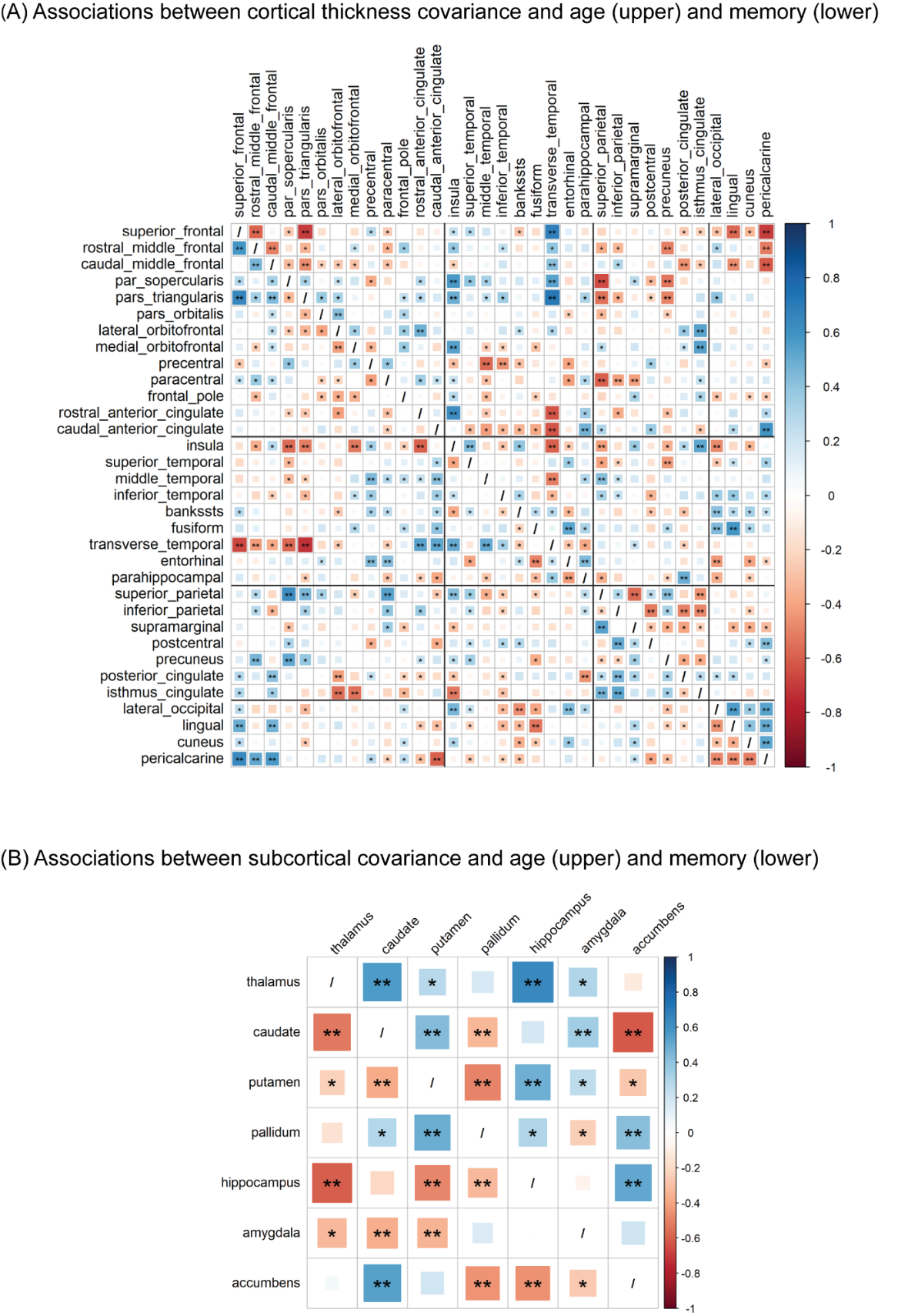


**Fig. S12**. Associations between structural covariance and age/memory in cortical thickness (A) and (B) subcortical volume. Covariance-age associations are shown in the upper right triangle and covariance-memory associations are shown in the lower left triangle.
